# Implications of non-uniqueness in phylogenetic deconvolution of bulk DNA samples of tumors

**DOI:** 10.1186/s13015-019-0155-6

**Published:** 2019-09-03

**Authors:** Yuanyuan Qi, Dikshant Pradhan, Mohammed El-Kebir

**Affiliations:** 1 Department of Computer Science, University of Illinois at Urbana-Champaign, Urbana, IL 61801 USA; 2 Department of Bioengineering, University of Illinois at Urbana-Champaign, Urbana, IL 61801 USA

**Keywords:** Phylogenetics, Intra-tumor heterogeneity, Inter-tumor heterogeneity, Somatic mutations, Single-nucleotide variant, Copy-number aberration, Structural variant, Metastasis, Evolution

## Abstract

**Background:**

Tumors exhibit extensive intra-tumor heterogeneity, the presence of groups of cellular populations with distinct sets of somatic mutations. This heterogeneity is the result of an evolutionary process, described by a phylogenetic tree. In addition to enabling clinicians to devise patient-specific treatment plans, phylogenetic trees of tumors enable researchers to decipher the mechanisms of tumorigenesis and metastasis. However, the problem of reconstructing a phylogenetic tree *T* given bulk sequencing data from a tumor is more complicated than the classic phylogeny inference problem. Rather than observing the leaves of *T* directly, we are given mutation frequencies that are the result of mixtures of the leaves of *T*. The majority of current tumor phylogeny inference methods employ the perfect phylogeny evolutionary model. The underlying Perfect Phylogeny Mixture (PPM) combinatorial problem typically has multiple solutions.

**Results:**

We prove that determining the exact number of solutions to the PPM problem is #P-complete and hard to approximate within a constant factor. Moreover, we show that sampling solutions uniformly at random is hard as well. On the positive side, we provide a polynomial-time computable upper bound on the number of solutions and introduce a simple rejection-sampling based scheme that works well for small instances. Using simulated and real data, we identify factors that contribute to and counteract non-uniqueness of solutions. In addition, we study the sampling performance of current methods, identifying significant biases.

**Conclusions:**

Awareness of non-uniqueness of solutions to the PPM problem is key to drawing accurate conclusions in downstream analyses based on tumor phylogenies. This work provides the theoretical foundations for non-uniqueness of solutions in tumor phylogeny inference from bulk DNA samples.

## Background

Cancer is characterized by somatic mutations that accumulate in a population of cells, leading to the formation of genetically distinct *clones* within the same tumor [1]. This *intra-tumor heterogeneity* is the main cause of relapse and resistance to treatment [2]. The evolutionary process that led to the formation of a tumor can be described by a *phylogenetic tree* whose leaves correspond to tumor cells at the present time and whose edges are labeled by somatic mutations. To elucidate the mechanisms behind tumorigenesis [2, 3] and identify treatment strategies [4, 5], we require algorithms that accurately infer a phylogenetic tree from DNA sequencing data of a tumor.

**Fig. 1:**
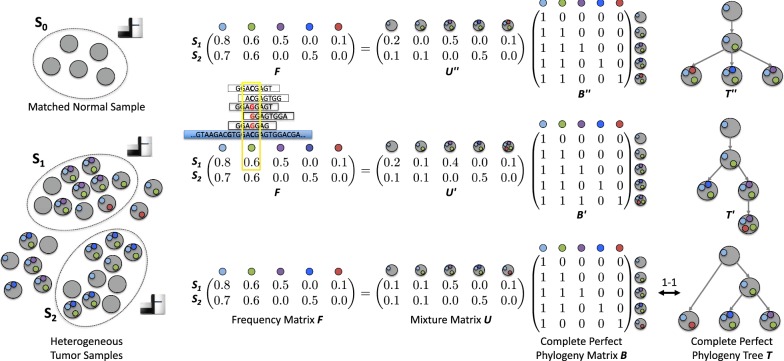
Overview of the Perfect Phylogeny Mixture (PPM) problem. By comparing the aligned reads obtained from bulk DNA sequencing data of a matched normal sample and *m* tumor samples, we identify *n* somatic mutations and their frequencies $F = \left[f_{p,c}\right]$. In the PPM problem, we are asked to factorize *F* into a mixture matrix *U* and a complete perfect phylogeny matrix *B*, explaining the composition of the *m* tumor samples and the evolutionary history of the *n* mutations present in the tumor, respectively. Typically, an input frequency matrix admits multiple distinct solutions. Here, matrix *F* has three solutions: (*U*, *B*), $(U',B')$ and $(U'',B'')$, where only (*U*, *B*) is the correct solution

Most cancer sequencing studies, including those from The Cancer Genome Atlas [6] and the International Cancer Genome Consortium [7], use bulk DNA sequencing technology, where samples are a mixture of millions of cells. While in classic phylogenetics, one is asked to infer a phylogenetic tree given its leaves, with bulk sequencing data we are asked to infer a phylogenetic tree given mixtures of its leaves in the form of mutation frequencies (Fig. 1). More specifically, one first identifies a set of loci containing somatic mutations present in the tumor by sequencing and comparing the aligned reads of a matched normal sample and one or more tumor samples. Based on the number reads of each mutation locus in a sample, we obtain *mutation frequencies* indicating the fraction of cells in the tumor sample that contain each mutation. From these frequencies, the task is to infer the phylogenetic tree under an appropriate evolutionary model that generated the data.

The most commonly used evolutionary model in cancer phylogenetics is the *two-state perfect phylogeny* model, where mutations adhere to the infinite sites assumption [8–16]. That is, for each mutation locus the actual mutation occurred exactly once in the evolutionary history of the tumor and was subsequently never lost. In practice, we construct a tumor phylogeny for mutation clusters rather than individual mutations. While the infinite sites assumption might be violated for individual mutations, a violation of this assumption for all the mutations in a cluster is rare. The underlying combinatorial problem of the majority of current methods is the Perfect Phylogeny Mixture (PPM) problem. Given an $m\times n$ frequency matrix *F*, we are asked to explain the composition of the *m* tumor samples and the evolutionary history of the *n* mutations. More specifically, we wish to factorize *F* into a mixture matrix *U* and a perfect phylogeny matrix *B*. Not only is this problem NP-complete [10], but multiple perfect phylogeny trees may be inferred from the same input matrix *F* (Fig. 1). Tumor phylogenies have been used to identify mutations that drive cancer progression [17, 18], to assess the interplay between the immune system and the clonal architecture of a tumor [19, 20] and to identify common evolutionary patterns in tumorigenesis and metastasis [21, 22]. To avoid any bias in such downstream analyses, all possible solutions must be considered. While non-uniqueness of solutions to PPM has been recognized in the field [11, 23], a rigorous analysis of its extent and consequences on sampling by current methods has been missing.

In this paper, we study the non-uniqueness of solutions to the PPM problem. On the negative side, we prove that the counting problem is #P-complete, hard to approximate within a constant factor and that it is hard sample to solutions uniformly at random (unless RP = NP). On the positive side, we give an upper bound on the number of solutions that can be computed in polynomial time, and introduce a simple rejection-based sampling scheme that samples solutions uniformly for modest numbers *n* of mutations. Using simulations and real data from a recent lung cancer cohort [18], we identify factors that contribute to non-uniqueness. In addition, we empirically study how the joint application of single-cell and long-read sequencing technologies with traditional bulk sequencing technology affects non-uniqueness. Finally, we find that current Markov chain Monte Carlo methods fail to sample uniformly from the solution space.

A preliminary version of this study was published as an extended abstract in RECOMB-CG [24].

## Preliminaries and problem statement

In this section, we review the Perfect Phylogeny Mixture problem, as introduced in [10] (where it was the called the Variant Allele Frequency Factorization Problem or VAFFP). As input, we are given a frequency matrix $F = \left[f_{p,c}\right]$ composed of allele frequencies of *n* single-nucleotide variants (SNVs) measured in *m* bulk DNA sequencing samples. In the following, we refer to SNVs as mutations. Each frequency $f_{p,c}$ indicates the proportion of cells in sample *p* that have mutation *c*.

### **Definition 1**

An $m \times n$ matrix $F = \left[f_{p,c}\right]$ is a *frequency matrix* provided ${f_{p,c} \in [0,1]}$ for all samples $p \in [m]$ and mutations $c \in [n]$.

The evolutionary history of all *n* mutations is described by a phylogenetic tree. We assume the absence of homoplasy—i.e. no back mutations and no parallel evolution—and define a complete perfect phylogeny tree *T* as follows.

### **Definition 2**

A rooted tree *T* on *n* vertices is a *complete perfect phylogeny tree* provided each edge of *T* is labeled with exactly one mutation from [*n*] and no mutation appears more than once in *T*.

We call the unique mutation $r \in [n]$ that does not label any edge of a complete perfect phylogeny tree *T* the *founder mutation*. Equivalently, we may represent a complete perfect phylogeny tree by an $n \times n$ binary matrix *B* subject to the following constraints.

### **Definition 3**

An $n \times n$ binary matrix $B = [b_{c,d}]$ is an *n-complete perfect phylogeny matrix* provided:

1. There exists exactly one $r \in [n]$ such that $\sum _{c=1}^n b_{r,c} = 1$.
2. For each $d \in [n] \setminus \{r\}$ there exists exactly one $c \in [n]$ such that $\sum _{e=1}^n b_{d,e} - \sum _{e=1}^n b_{c,e} = 1$, and $b_{d,e} \ge b_{c,e}$ for all $e \in [n]$.
3. $$b_{c,c} = 1$$ for all $$c \in [n]$$.

These three conditions correspond to distinctive features in complete perfect phylogenetic trees. Condition 1 states the existence of a single root vertex. Condition 2 indicates that any mutation *d* other than the root has a unique parent *c*. Condition 3 removes symmetry to ensure a one-to-one correspondence between complete perfect phylogeny matrices and complete perfect phylogenetic trees.

While the rows of a perfect phylogeny matrix *B* correspond to the leaves of a perfect phylogeny tree *T* (as per Definition 1), a *complete* perfect phylogeny matrix *B* includes all vertices of *T*. The final ingredient is an $m \times n$ mixture matrix *U* defined as follows.

### **Definition 4**

An $m \times n$ matrix $U = [u_{p,c}]$ is a *mixture matrix* provided ${u_{p,c} \in [0,1]}$ for all samples $p \in [m]$ and mutations $c \in [n]$, and $\sum _{c=1}^n u_{p,c} \le 1$ for all samples $p \in [m]$.

Each row of *U* corresponds to a bulk sample whose entries indicate the fractions of the corresponding clones represented by the rows in *B*. Since we omit the normal clone (not containing any mutations), each row of *U* sums up to at most 1, the remainder being the fraction of the normal clone in the sample. Thus, the forward problem of obtaining a frequency matrix *F* from a complete perfect phylogeny matrix *B* and mixture matrix *U* is trivial. That is, $F = UB$. We are interested in the inverse problem, which is defined as follows.

### **Problem 1**

(*Perfect* *Phylogeny* *Mixture* *(PPM)*) Given a frequency matrix *F*, find a complete perfect phylogeny matrix *B* and mixture matrix *U* such that $F = UB$.

El-Kebir et al. [10] showed that a solution to PPM corresponds to a constrained spanning arborescence of a directed graph $G_F$ obtained from *F*, as illustrated in Additional file 1: Figure S2. This directed graph $G_F$ is called the *ancestry graph* and is defined as follows.

### **Definition 5**

The *ancestry graph* $G_F$ obtained from frequency matrix $F = \left[f_{p,c}\right]$ has *n* vertices $V(G_F) = \{1,\ldots ,n\}$ and there is a directed edge $(c,d) \in E(G_F)$ if and only if $f_{p,c} \ge f_{p,d}$ for all samples $p \in [m]$.

As shown in [10], the square matrix *B* is invertible and thus matrix *U* is determined by *F* and *B*. We denote the set of children of the vertex corresponding to a mutation $c \in [n] \setminus \{r\}$ by $\delta (c)$, and we define $\delta (r) = \{r(T)\}$.

### **Proposition 1**

(Ref. [10]) *Given frequency matrix* $F = \left[f_{p,c}\right]$ *and complete perfect phylogeny matrix* $B = [b_{c,d}],$ *matrix* $U = [u_{p,c}]$ *where* $u_{p,c} = f_{p,c} - \sum _{d \in \delta (c)} f_{p,d}$ *is the unique matrix U such that* $F = UB.$

For matrix *U* to be a mixture matrix, it is necessary and sufficient to enforce non-negativity as follows.

### **Theorem 2**

(Ref. [10]) *Let* $F = \left[f_{p,c}\right]$ *be a frequency matrix and* $G_F$ *be the corresponding ancestry graph. Then, complete perfect phylogeny matrix B* *and associated matrix U are a solution to* PPM *instance F* *if and only if B* *T of* $G_F$ satisfying

SC $$\begin{aligned} f_{p,c} \ge \sum _{d \in \delta _{\text {out}}(c)} f_{p,d} \quad \forall p \in [m], c \in [n]. \end{aligned}$$

The above inequality is known as the sum condition (SC), requiring that each mutation has frequency greater than the sum of the frequencies of its children in all samples. In this equation, $\delta _\text {out}(c)$ denotes the set of children of vertex *c* in rooted tree *T*. A *spanning arborescence* *T* of a directed graph $G_F$ is defined as a subset of edges that induce a rooted tree that spans all vertices of $G_F$.

**Fig. 2:**
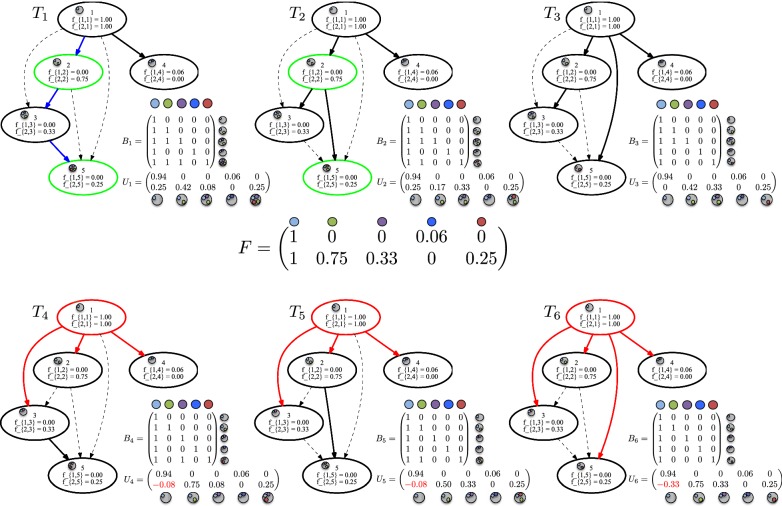
Example PPM instance *F* has three solutions. Frequency matrix *F* corresponds to a simulated $n=5$ instance (#9) and has $m=2$ samples. The ancestry graph $G_F$ has six spanning arborescences. Among these, only trees $T_1$, $T_2$ and $T_3$ satisfy the sum condition (SC), whereas trees $T_4$, $T_5$ and $T_6$ violate (SC) leading to negative entries in $U_4$, $U_5$ and $U_6$. Tree $T_1$ is the simulated tree of this instance. Trees $T_2$ and $T_3$ differ from $T_1$ by only one edge, and thus each have an edge recall of $3/4 = 0.75$

While finding a spanning arborescence in a directed graph can be done in linear time (e.g., using a depth-first or breadth-first search), the problem of finding a spanning arborescence in $G_F$ adhering to (SC) is NP-hard [10, 23]. Moreover, the same input frequency matrix *F* may admit more than one solution (Fig. 2).

## Methods

We start by giving a combinatorial characterization of solutions to the PPM problem (“Characterization of the solution space” section), followed by a complexity analysis of the counting and sampling version #PPM (“Complexity” section). “Additional constraints on the solution space” section describes additional constraints that reduce the number of solutions. Finally, “Uniform sampling of solutions” section introduces a rejection sampling scheme that is able to sample uniformly at random.

### Characterization of the solution space

Let *F* be a frequency matrix and let $G_F$ be the corresponding ancestry graph. By Theorem 2, we have that solutions to the PPM instance *F* are spanning arborescences *T* in the ancestry graph $G_F$ that satisfy (SC). In this section, we describe additional properties that further characterize the solution space. We start with the ancestry graph $G_F$.

#### **Fact 3**

*If there exists a path from vertex c to vertex* *d then* $(c,d) \in E(G_F).$

A pair of mutations that are not connected by a path in $G_F$ correspond to two mutations that must occur on distinct branches in any solution. Such pairs of incomparable mutations are characterized as follows.

#### **Fact 4**

*Ancestry graph* $G_F$ *does not contain the edge (c,* *d) nor the edge* *(d,* *c) if and only if there exist two samples* $p,q \in [m]$ *such that* $f_{p,c} > f_{p,d}$ *and* $f_{q,c} < f_{q,d}.$

We define the branching coefficient as follows.

#### **Definition 6**

The *branching coefficient* $\gamma (G_F)$ is the fraction of unordered pairs (*c*, *d*) of distinct mutations such that $(c,d) \not \in E(G_F)$ and $(d,c) \not \in E(G_F)$.

In the single-sample case, where frequency matrix *F* has $m=1$ sample, we have that $\gamma (G_F) = 0$. This is because either $f_{1,c} \ge f_{1,d}$ or $f_{1,d} \ge f_{1,c}$ for any ordered pair (*c*, *d*) of distinct mutations. Since an arborescence is a rooted tree, we have the following fact.

#### **Fact 5**

*For* $G_F$ *to contain a spanning arborescence there must exist a vertex in* $G_F$ *from which all other vertices are reachable.*

Note that $G_F$ may contain multiple source vertices from which all other vertices are reachable. Such source vertices correspond to repeated columns in *F* whose entries are greater than or equal to every other entry in the same row. In most cases the ancestry graph $G_F$ does not contain any directed cycles because of the following property.

#### **Fact 6**

*Ancestry graph* $G_F$ *is a directed acyclic graph (DAG) if and only if F has no repeated columns.*

In the case where $G_F$ is a DAG and contains at least one spanning arborescences, we know that all spanning arborescence *T* of $G_F$ share the same root vertex. This root vertex *r* is the unique vertex of $G_F$ with in-degree 0.

#### **Fact 7**

*If* $G_F$ *is a DAG and contains a spanning arborescence then there exists exactly one vertex r in* $G_F$ *from which all other vertices are reachable.*

Figure 2 shows the solutions to a PPM instance *F* with $m=2$ tumor samples and $n=5$ mutations. Since *F* has no repeated columns, the corresponding ancestry graph $G_F$ is a DAG. Vertex $r = 1$ is the unique vertex of $G_F$ without any incoming edges. There are three solutions to *F*, i.e. $T_1$, $T_2$ and $T_3$ are spanning arborescences of $G_F$, each rooted at vertex $r = 1$ and each satisfying (SC). How do we know that *F* has three solutions in total? This leads to the following problem.

#### **Problem 2**

(*#-Perfect* *Phylogeny* *Mixture (#PPM)*) Given a frequency matrix *F*, count the number of pairs (*U*, *B*) such that *B* is a complete perfect phylogeny matrix, *U* is a mixture matrix and $F = UB$.

Since solutions to *F* correspond to a subset of spanning arboscences of $G_F$ that satisfy (SC), we have the following fact.

#### **Fact 8**

*The number of solutions to a PPM instance F is at most the number of spanning arborescences in the ancestry graph* $G_F.$

Kirchhoff’s elegant matrix tree theorem [25] uses linear algebra to count the number of spanning trees in a simple graph. Tutte extended this theorem to count spanning arborescences in a directed graph $G = (V,E)$ [26]. Briefly, the idea is to construct the $n \times n$ Laplacian matrix $L = [\ell _{i,j}]$ of *G*, where

1 $$\begin{aligned} \ell _{i,j} = {\left\{ \begin{array}{ll} \mathrm {deg}_{\text {in}}(j), &{} \text{ if } i = j,\\ -1, &{} \text { if }i \ne j\text { and }(i,j) \in E\\ 0, &{} \text{ otherwise. } \end{array}\right. } \end{aligned}$$

Then, the number of spanning arborescences $N_i$ rooted at vertex *i* is $\text {det}({\hat{L}}_i)$, where ${\hat{L}}_i$ is the matrix obtained from *L* by removing the *i*-th row and column. Thus, the total number of spanning arborescences in *G* is $\sum _{i=1}^n \text {det}({\hat{L}}_i)$.

By Fact 6, we have that $G_F$ is a DAG if *F* has no repeated columns. In addition, by Fact 7, we know that $G_F$ must have a unique vertex *r* with no incoming edges. We have the following technical lemma.

#### **Lemma 9**

*Let* $G_F$ *be a DAG and let* $r(G_F)$ *be its unique source vertex. Let* $\pi$ *be a topological ordering of the vertices of* $G_F.$ *Let* $L'=[\ell '_{i,j}]$ *be the matrix obtained from* $L = [\ell _{i,j}]$ *by permuting its rows and columns according to* $\pi,$ *i.e.* $\ell '_{i,j} = \ell _{\pi (i),\pi (j)}.$ *Then,* $L'$ *is an upper triangular matrix and* $\pi (1) = r(G_F).$

#### *Proof*

Assume for a contradiction that $L'$ is not upper triangular. Thus, there must exist vertices $i,j \in [n]$ such that $j > i$ and $\ell '_{j,i} \ne 0$. By definition of *L* and $L'$, we have that $\ell '_{j,i} = -1$. Thus $(\pi (j),\pi (i)) \in E(G_F)$, which yields a contradiction with $\pi$ being a topological ordering of $G_F$. Hence, $L'$ is upper triangular. From Fact 7 it follows that $\pi (1) = r(G_F)$. $\square$

Since the determinant of an upper triangular matrix is the product of its diagonal entries, it follows from the previous lemma that $\text {det}({\hat{L}}'_1) = \prod _{i=1}^{n-1} {\hat{\ell }}'_{i,i}$. Combining this fact with Tutte’s directed matrix-tree theorem, yields the following result.

#### **Theorem 10**

*Let F* *be a frequency matrix without any repeated columns and let r be the unique mutation such that* $f_{p,r} \ge f_{p,c}$ *for all mutations c and samples* *p*. *Then the number of solutions to F is at most the product of the in-degrees of all vertices* $c \ne r$ *in* $G_F.$

In Fig. 2, the number of spanning arborescences in $G_F$ is $\text {deg}_{\text {in}}(2) \cdot \text {deg}_{\text {in}}(3) \cdot \text {deg}_{\text {in}}(4) \cdot \text {deg}_{\text {in}}(5) = 1 \cdot 2 \cdot 1 \cdot 3 = 6$. To compute the number of spanning arborescences of $G_F$ that satisfy (SC), we can simply enumerate all spanning arborescences using, for instance, the Gabow-Myers algorithm [27] and only output those that satisfy (SC). El-Kebir et al. [23] extended this algorithm such that it maintains (SC) as an invariant while growing arborescences. Applying both algorithms on the instance in Fig. 2 reveals that trees $T_1$, $T_2$ and $T_3$ comprise all solutions to *F*. We note that the enumeration algorithm in [23] has not been shown to be an output-sensitive algorithm.

### Complexity

Deciding whether a frequency matrix *F* can be factorized into a complete perfect phylogeny matrix *B* and a mixture matrix *U* is NP-complete [10] even in the case where $m=2$ [23]. We showed this by reduction from SubsetSum, defined as follows.

#### **Problem 3**

(*SubsetSum*) Given a set of unique positive integers *S*, and a positive integer $t < \sum _{s \in S}s$, find a subset *D* of *S* such that $\sum _{s \in D}s=t$.

As such, the corresponding counting problem #PPM is NP-hard. Here, we prove a stronger result, i.e. #PPM is #P-complete.

#### **Theorem 11**

*#PPM* *is #P-complete even when* $m = 2$.

To understand this result, recall the complexity class NP. This class is composed of decision problems that have *witnesses* that can be verified in polynomial time. The complexity class #P consists of counting problem that are associated with decision problems in NP. That is, rather than outputting yes/no for a given instance, we are interested in the number of witnesses of the instance. The class #P-complete is similarly defined as NP-complete and is composed of the hardest counting problems in #P. That is, if one #P-complete problem is solvable in polynomial time then all problems in #P are solvable in polynomial time. How do we show that a counting problem $\#Y$ is #P-complete? To do so, we need to show two things. First, we need to show that the underlying decision problem is in NP. Second, we need to show that another #P-complete problem $\#X$ is just as hard as $\#Y$. One way of showing this is using a polynomial-time parsimonious reduction from $\#X$ to $\#Y$, defined as follows.

#### **Definition 7**

Let *X* and *Y* be decision problems in NP, and let $\#X$ and $\#Y$ be the corresponding counting problems. Let $\Sigma ^*$ $(\Pi ^*)$ be the set of instances of *X* (*Y*). Given instances $x \in \Sigma ^*$ and $y \in \Pi ^*$, let *X*(*x*) and *Y*(*y*) be the corresponding set of witnesses. A reduction $\sigma :\Sigma ^* \rightarrow \Pi ^*$ from $\#X$ to $\#Y$ is *parsimonious* if $|X(x)|=|Y(\sigma (x))|$ and $\sigma (x)$ can be computed in time polynomial in |*x*| for all $x\in \Sigma ^*$.

We prove Theorem 11 in two steps by considering the counting version #SubsetSum of SubsetSum. First, we show that #SubsetSum is #P-complete by giving a parsimonious reduction from #Mono-1-in-3SAT, a known #P-complete problem [28].

#### **Lemma 12**

*There exists a parsimonious reduction from #Mono-1-*in*-3SAT* *to #* *S*ubset*Sum*.

#### *Proof*

See Additional file 1. $\square$

Second, we show that the previously used reduction to prove NP-completeness [23] from SubsetSum of PPM is also a parsimonious reduction.

#### **Lemma 13**

*There exists a parsimonious reduction from #S* *ubset* *S* *um* *to #PPM* *restricted to* $m=2$ *samples.*

#### *Proof*

See Additional file 1. $\square$

Combining these two results yields the theorem. One way to deal with this hardness result is to resort to approximation algorithms. In particular, for counting problems the following randomized approximation algorithms are desirable.

#### **Definition 8**

(*Ref.* [29]) A *fully polynomial randomized approximation scheme* (FPRAS) for a counting problem is a randomized algorithm that takes as input an instance *x* of the problem and error tolerance $\varepsilon >0$, and outputs a number $N'$ in time polynomial in $1/\varepsilon$ and |*x*| such that $\Pr \left[(1+\varepsilon )^{-1} N \le N' \le (1+\varepsilon )N\right]\ge 0.75$, where *N* is the answer to the counting problem.

Suppose we have an FPRAS for #PPM. What would the implications be? Recall the complexity class RP, which is composed of decision problems that admit randomized polynomial time algorithms that return no if the correct answer is no and otherwise return yes with probability at least 1/2. We can use the FPRAS for PPM to construct a randomized polynomial time algorithm for the decision problem PPM, returning yes if the FPRAS gives a non-zero output, and returning no otherwise. Obviously, this algorithm is always correct for no-instances, and returns the correct result at least 75% of the times for yes-instances. Since PPM is NP-complete, this would imply that RP = NP.

#### **Corollary 14**

*There exists no FPRAS for #PPM* *unless RP = NP.*

Regarding the sampling problem of PPM, it would be desirable to sample solutions almost uniformly at random, which can be achieved by the following set of algorithms.

#### **Definition 9**

(*Ref.* [29]) A *fully-polynomial almost uniform sampler* (FPAUS) for a sampling problem is a randomized algorithm that takes as input an instance *x* of the problem and a sampling tolerance $\delta >0$, and outputs a solution in time polynomial in |*x*| and $\log \delta ^{-1}$ such that the difference of the probability distribution of solutions output by the algorithm and the uniform distribution on all solutions is at most $\delta$.

However, the existence of an FPAUS to sample the solutions of PPM would similarly imply that RP=NP (i.e. setting $\delta \le 0.5$).

#### **Corollary 15**

*There exists no FPAUS to sample solutions of PPM* *unless RP = NP.*

### Additional constraints on the solution space

*Long-read sequencing* Most cancer sequencing studies are performed using next-generation sequencing technology, producing short reads containing between 100 and 1000 basepairs. Due to the small size of short reads, it is highly unlikely to observe two mutations that occur on the same read (or read pair). With (synthetic) long read sequencing technology, including 10× Genomics, Pacbio and Oxford Nanopore, one is able to obtain reads with millions of basepairs. Thus, it becomes possible to observe long reads that contain more than one mutation.

As described in [30], the key insight is that a pair (*c*, *d*) of mutations that occur on the same read orginate from a single DNA molecule of a single cell, and thus *c* and *d* must occur on the same path in the phylogenetic tree. Such mutation pairs provide very strong constraints to the PPM problem. For example in Fig. 2, in addition to frequency matrix *F*, we may be given that mutations 2 and 5 have been observed on a single read. Thus, in $T_1$ and $T_2$ the pair is highlighted in green because it is correctly placed on the same path from the root on the inferred trees. However, the two mutations occur on distinct branches on $T_3$, which is therefore ruled out as a possible solution.

*Single-cell sequencing* With single-cell sequencing, we are able to identify the mutations that are present in a single tumor cell. If in addition to bulk DNA sequencing samples, we are given single cell DNA sequencing data from the same tumor, we can constrain the solution space to PPM considerably. In particular, each single cell imposes that its comprising mutations must correspond to a connected path in the phylogenetic tree. These constraints have been described recently in [31].

For an example of these constraints, consider frequency matrix *F* described in Fig. 2. In addition to frequency matrix *F*, we may observe a single cell with mutations $\{1,2,3,5\}$. $T_1$ is the only potential solution as this is the only tree which places all four mutations on a single path, highlighted in blue. Trees $T_2$ and $T_3$ would be ruled out because the mutation set $\{1,2,3,5\}$ does not induce a connected path in these two trees.

We note that the constraints described above for single-cell sequencing and long-read sequencing assume error-free data. In practice, one must incorporate an error model and adjust the constraints accordingly. However, the underlying principles will remain the same.

### Uniform sampling of solutions

Typically, the number *m* of bulk samples equals 1, but there exist multi-region datasets where *m* may be up to 10. On the other hand, the number *n* of mutations ranges from 10 to 1000. In particular, for solid tumors in adults we typically observe thousands of point mutations in the genome. As such, exhaustive enumeration of solutions is infeasible in practice. To account for non-uniqueness of solutions and to identify common features shared among different solutions, it would be desirable to have an algorithm that samples uniformly from the solution space. However, as the underlying decision problem is NP-complete, the problem of sampling uniformly from the solution space for arbitrary frequency matrices *F* is NP-hard. Thus, one must resort to heuristic approaches.

One class of such approaches employs Markov chain Monte Carlo (MCMC) for sampling from the solution space [9, 14, 15]. Here, we describe an alternative method based on rejection sampling. This method is guaranteed to sample uniformly from the solution space. Briefly, the idea is to generate a spanning arborescence *T* from $G_F$ uniformly at random and then test whether *T* satisfies (SC). In the case where *T* satisfies (SC), we report *T* as a solution and otherwise reject *T*.

For the general case where $G_F$ may have a directed cycle, we use the cycle-popping algorithm of Propp and Wilson [32]. Note that this only happens when there are mutations with identical frequencies across all samples, i.e. identical columns in the frequency matrix *F*. This algorithm generates a uniform spanning arborescence in time $O(\tau ({\tilde{G}}_F))$ where $\tau ({\tilde{G}}_F)$ is the expected hitting time of ${\tilde{G}}_F$. More precisely, ${\tilde{G}}_F$ is the multi-graph obtained from $G_F$ by including self-loops such that the out-degrees of all its vertices are identical.

For the case where $G_F$ is a DAG with a unique source vertex *r*, there is a much simpler sampling algorithm. We simply assign each vertex $c \ne r$ to a parent $\pi (c) \in \delta _{\text {in}}(c)$ uniformly at random. It is easy to verify that the resulting function $\pi$ encodes a spanning arborescence of $G_F$. Thus, the running time of this procedure is $O(E(G_F))$. In both cases, the probability of success equals the fraction of spanning arborescences of $G_F$ that satisfy (SC) among all spanning arborescences of $G_F$.

An implementation of the rejection sampling for the case where $G_F$ is a DAG is available on https://github.com/elkebir-group/OncoLib.

## Results

Figures 1 and 2 show anecdotal examples of non-uniqueness of solutions to the Perfect Phylogeny Mixture problem. The following questions arise: is non-uniqueness a widespread phenomenon in PPM instances? Which factors contribute to non-uniqueness and how does information from long-read sequencing and single-cell sequencing reduce non-uniqueness? Finally, are current MCMC methods able to sample uniformly from the space of solutions?

To answer these questions, we used real data from a lung cancer cohort [18] and simulated data generated by a previously published tumor simulator [33]. For the latter, we generated 10 complete perfect phylogeny trees $T^*$ for each number $n \in \{3,5,7,9,11,13\}$ of mutations. The simulator assigned each vertex $v \in V(T^*)$ a frequency $f(v) \ge 0$ such that $\sum _{v \in V(T^*)} f(v) = 1$. For each simulated complete perfect phylogeny tree $T^*$, we generated $m \in \{1,2,5,10\}$ bulk samples by partitioning the vertex set $V(T^*)$ into *m* disjoint parts followed by normalizing the frequencies in each sample. This yielded a frequency matrix *F* for each combination of *n* and *m*. In total, we generated $10 \cdot 6 \cdot 4 = 240$ instances (Additional file 1: Tables S1–S7). The data and scripts to generate the results are available on https://github.com/elkebir-group/PPM-NonUniq.

### What contributes to non-uniqueness?

In both real and simulated data, we find that the two main factors that influence non-uniqueness are the number *n* of mutations and the number *m* of samples taken from the tumor. The former contributes to non-uniqueness while the latter reduces it, as we will show in the following.

**Fig. 3:**
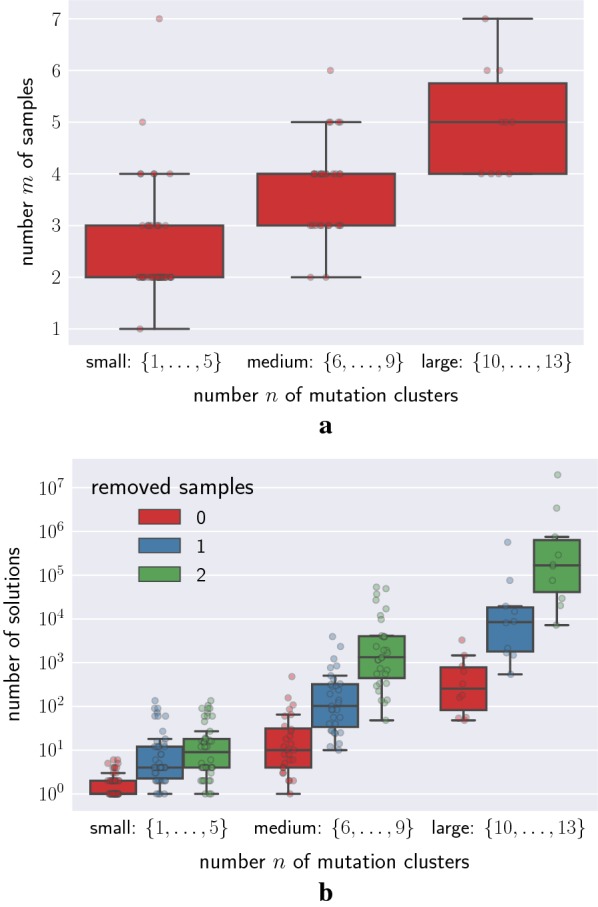
Non-uniqueness of solutions in a multi-region lung cancer cohort of 100 patients [18]. **a** In this lung cancer cohort of 100 patients, 1 to 7 regional samples (*y*-axis) of each cancer have undergone bulk DNA sequencing, followed by the identification of mutations clusters (*x*-axis) using PyClone [34]. **b** This dataset exhibits extensive non-uniqueness of solutions (median of 3 solutions per patient). The number of solutions increased when downsampling the number *m* of bulk samples (color indicates the number of removed samples)

We considered a lung cancer cohort of 100 patients [18], where tumors have undergone multi-region bulk DNA sequencing. Subsequently, the authors used PyClone [34] to cluster mutations with similar cancer cell fractions. The number *n* of mutation clusters varied from 2 to 13 clusters and the number *m* of samples varied from 1 to 7 (Fig. 3a). To account for uncertainty in mutation cluster frequencies, we consider a 90% confidence interval obtained from the cancer cell fractions of clustered mutations and solve an interval version of the PPM problem (described in Ref. [23]). To see how the number *m* of bulk samples affects the number of solutions, we downsample by randomly removing 1 or 2 samples. We find that this dataset exhibits extensive non-uniqueness of solutions, with the number of solutions ranging from 1 to 3280 (Fig. 3b and Additional file 1: Table S1 and S2). We find that the number of solutions increased with increasing number *n* of mutation clusters, whereas it decreased when downsampling the number *m* of samples (Fig. 3b).

**Fig. 4:**
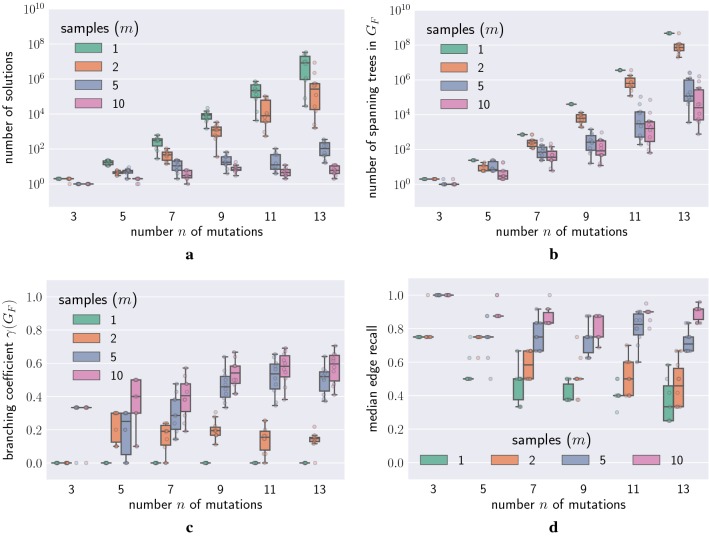
Factors that contribute to non-uniqueness. **a** The number of solutions increased with increasing number *n* of mutations, but decreased with increasing number *m* of bulk samples. **b** Every solution of an PPM instance *F* is a spanning arborescence in the ancestry graph $G_F$. The number of spanning arborescences in $G_F$ also increased with increasing *n* and decreased with increasing *m*. **c** The decrease in the number of solutions and spanning arborescences with increasing *m* is explained by the branching coefficient of $\gamma (G_F)$, which is the fraction of distinct pairs of mutations that occur on distinct branches in $G_F$. The fraction of such pairs increased with increasing *m*. **d** The median edge recall of the inferred trees *T* increased with increasing *m*

We observed similar trends in simulated data. That is, as we increased the number *n* of mutations from 3 to 13 in our simulations, we observed that the number of solutions increased exponentially (Fig. 4a). On the other hand, the number *m* of samples had an opposing effect: with increasing *m* the number of solutions decreased.

To understand why we observed these two counteracting effects, we computed the number of spanning arborescences in each ancestry graph $G_F.$ Figure 4b shows that the number of spanning arborescences exhibited an exponential increase with increasing number *n* of mutations, whereas increased number *m* of samples decreased the number of spanning arborescences. The latter can be explained by studying the effect of the number *m* of samples on the branching coefficient $\gamma (G_F)$. Figure 4c shows that the branching coefficient increased with increasing *m*, with branching coefficient $\gamma (G_F) = 0$ for all $m=1$ instances *F*. This finding illustrates that additional samples reveal branching of mutations. That is, in the case where $m=1$ one does not observe branching in $G_F$, whereas as $m \rightarrow \infty$ each sample will be composed of a single cell with binary frequencies and the ancestry graph $G_F$ will be a rooted tree.

Adding mutations increases the complexity of the problem, as reflected by the number of solutions. To quantify how distinct each solution *T* is to the simulated tree $T^*$, we computed the edge recall of *T* defined as $|E(T) \cap E(T^*)| / |E(T^*)|$ (note that $|E(T^*)| = n - 1$ by definition). A recall value of 1 indicates that the inferred tree *T* is identical to the true tree $T^*$. Figure 4d shows that the median recall decreased with increasing number *n* of mutations. However, as additional samples provide more information, the recall increased with increasing number *m* of samples.

### How to reduce non-uniqueness?

As discussed in “Additional constraints on the solution space” section, the non-uniqueness of solutions can be reduced through various sequencing techniques such as single-cell sequencing and long-read sequencing. We considered the effect of both technologies on the $n=9$ instances (Additional file 1: Table S6).

**Fig. 5:**
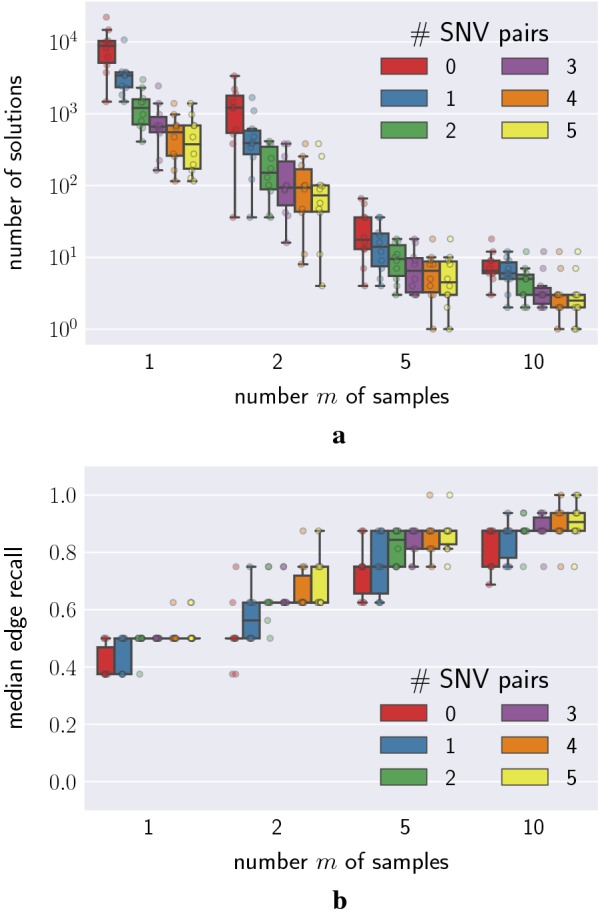
Long-read sequencing reduces the size of the solution space. **a** The number of solutions decreased with increasing pairs of mutations that occurred on the same read. **b** The median edge recall increased with increasing pairs of mutations that co-occur on a read

By taking longer reads of the genome, long-read sequencing can identify mutations which coexist in a clone if they appear near one another on the genome. If two mutations are observed together on a long read, then one mutation is ancestral to the other. That is, on the true phylogenetic tree $T^*$ there must exist a path from the root to a leaf containing both mutations. We varied the number of mutation pairs observed together from 0 to 5 and observed that increasing this number reduced the size of the solution space (Fig. 5a). In addition, incorporating more simulated long-read information resulted in increased recall of the inferred trees (Fig. 5b).

**Fig. 6:**
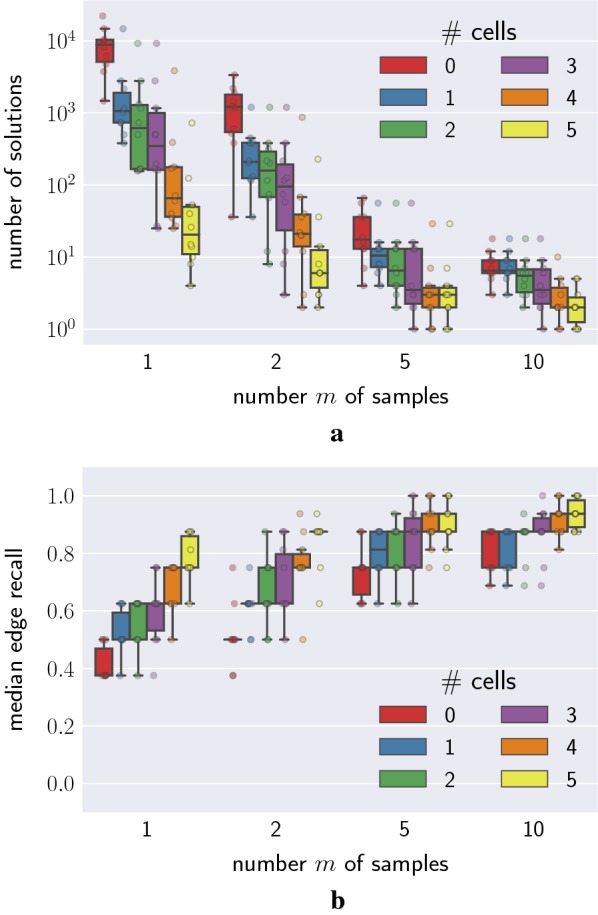
Joint bulk and single-cell sequencing reduces the size of the solution space. **a** The number of solutions decreased with increasing number of single cells. **b** The median edge recall increased with increasing number of single cells

Single-cell sequencing illuminates all of the mutations present in a single clone in a tumor. This reveals a path from the root of the true phylogenetic tree $T^*$ down to a leaf. Fig. 6a shows the effect that single-cell sequencing has on the size of the solution space. We found that, as we increased the number of known paths (sequenced single cells) in the tree from 0 to 5, the solution space decreased exponentially. Additionally, the inferred trees were more accurate with more sequenced cells, as shown in Fig. 6b by the increase in median edge recall. These effects are more pronounced when fewer samples are available.

In summary, while both single-cell and long-read sequencing reduce the extent of non-uniqueness in the solution space, single-cell sequencing achieves a larger reduction than long-read sequencing.

### How does non-uniqueness affect current methods?

To study the effect of non-uniqueness, we considered two current methods, PhyloWGS [14] and Canopy [15], both of which use Markov chain Monte Carlo to sample solutions from the posterior distribution. Rather than operating from frequencies $F = \left[f_{p,c}\right]$, these two methods take as input two integers $a_{p,c}$ and $d_{p,c}$ for each mutation *c* and sample *p*. These two integers are, respectively, the number of reads with mutation *c* and the total number of reads. Given $A = [a_{p,c}]$ and $D = [d_{p,c}]$, PhyloWGS and Canopy aim to infer a frequency matrix ${\hat{F}}$ and phylogenetic tree *T* with maximum data likelihood $\Pr (D,A \mid {\hat{F}})$ such that *T* satisfies (SC) for matrix ${\hat{F}}$. In addition, the two methods cluster mutations that are inferred to have similar frequencies across all samples. To use these methods in our error-free setting, where we are given matrix $F = \left[f_{p,c}\right]$, we set the total number of reads for each mutation *c* in each sample *p* to a large number, i.e. $d_{p,c} = 1,000,000$. The number of variant reads is simply set as $a_{p,c} = f_{p,c} \cdot d_{p,c}$. Since both PhyloWGS and Canopy model variant reads $a_{p,c}$ as draws from a binomial distribution parameterized by $d_{p,c}$ and ${\hat{f}}_{p,c}$, the data likelihood is maximized when ${\hat{F}} = F$. We also discard generated solutions where mutations are clustered. Hence, we can use these methods in the error-free case.

We ran PhyloWGS, Canopy, and our rejection sampling method (“Uniform sampling of solutions” section) on all $n=7$ instances (Additional file 1: Table S5). We used the default settings for PhyloWGS (2500 MCMC samples, burnin of 1000) and Canopy (burnin of 100 and 1 out of 5 thinning), with 20 chains per instance for PhyloWGS and 15 chains per instance for Canopy. For each instance, we ran the rejection sampling algorithm until it generated 10,000 solutions that satisfy (SC).

**Fig. 7:**
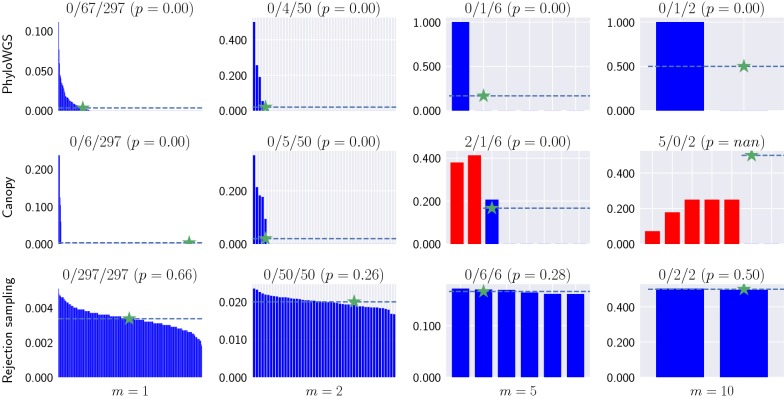
PhyloWGS and Canopy do not sample uniformly from the solution space. We consider an $n=7$ instance (#81) with varying number $m \in \{1,2,5,10\}$ of bulk samples (columns), from which we sample solutions using different methods (rows). Each plot shows the relative frequency (*y*-axis) of identical trees (*x*-axis) output by each method, with the simulated tree indicated by ‘$\star$’. While blue bars are correct solutions [satisfying (SC)], red bars correspond to incorrect solutions [violating (SC)]. Dashed line indicates the expected relative frequency in the case of uniformity. The title of each plot lists the number of incorrect solutions, the number of recovered correct solutions, the total number of correct solutions and the *p*-value of the chi-squared test of uniformity (null hypothesis is that the samples come from a uniform distribution)

Figure 7 shows one $n=7$ instance (#81) with varying number $m \in \{1,2,5,10\}$ of samples. For this instance, all the trees output by PhyloWGS satisfied the sum condition. However, the set of solutions was not sampled uniformly, with only 67 out 297 trees generated for $m=1$ samples. For $m=5$, this instance had six unique solutions, with PhyloWGS only outputting trees that corresponded to a single solution among these six solutions (Additional file 1: Fig. S5). Similarly, Canopy failed to sample solutions uniformly at random. In addition, Canopy failed to recover any of the two $m=10$ solutions and recovered incorrect solutions for $m=5$. The rejection sampling method recovered all solutions for each value of *m*. In addition, we performed a Chi-square goodness of fit test comparing the distribution of trees generated by rejection sampling to the uniform distribution. The large *p*-values indicate that the rejection sampling procedure sampled solutions uniformly at random. Additional file 1: Figures S6–S8 show similar patterns for the other $n=7$ instances.

There are two possible factors contributing to the non-uniformity of the sampling results of PhyloWGS and Canopy. First, the Tree-Structured Stick Breaking (TSSB) process used by PhyloWGS to generate the tree topology does not give a uniform prior over the space of trees. Second, the two MCMC algorithms might not converge onto the stationary distribution in reasonable time. Indeed, by our hardness result for the sampling problem of PPM (Corollary 15), we expect the mixing time to grow exponentially with increasing number *n* of mutations and increasing number *m* of samples.

**Fig. 8:**
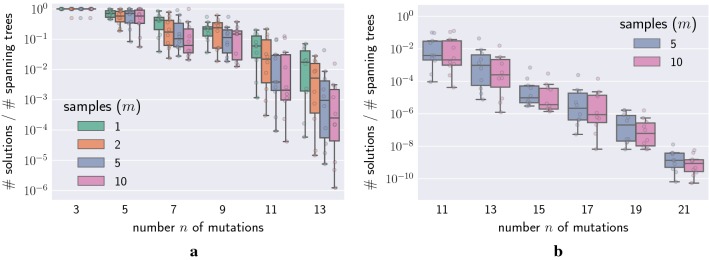
Although rejection sampling achieves uniformity, it becomes impractical with increasing number *n* of mutations. **a** Plot shows the ratio of the number of solutions to spanning arborescences. Observe that the number of spanning trees increased with the number *n* of mutations far more rapidly than the number of solutions. **b** With further increases in *n*, the ratio rapidly decreased and the odds of randomly sampling a solution from the space of spanning arborescences becomes infeasible

Given a frequency matrix *F*, the success probability of the rejection sampling approach equals the fraction between the number of solutions and the number of spanning arborescences in $G_F$, as shown empirically in Additional file 1: Table S9. As such, this approach does not scale with increasing *n*. Indeed, Fig. 8a shows that the fraction of spanning trees which also fulfill the sum condition is initially high when the number of mutations is low. With $n=11$ mutations, the fraction is approximately $10^{-2}$ and rejection sampling can be considered to be feasible. However, as the number of mutations is increased further, rejection sampling become infeasible as the fraction can drop to $10^{-10}$ for $n=21$ mutations (Fig. 8b). Therefore, a better sampling approach is required.

## Conclusions

In this work, we studied the problem of non-uniqueness of solutions to the Perfect Phylogeny Mixture (PPM) problem. In this problem, we are given a frequency matrix *F* that determines a directed graph $G_F$ called the ancestry graph. The task is to identify a spanning arborescence *T* of $G_F$ whose internal vertices satisfy a linear inequality whose terms are entries of matrix *F*. We formulated the #PPM problem of counting the number of solutions to an PPM instance. We proved that the counting problem is #P-complete and that no FPRAS exists unless RP = NP. In addition we argued that no FPAUS exists for the sampling problem unless RP = NP. On the positive side, we showed that the number of solutions is at most the number of spanning arborescences in $G_F$, a number that can be computed in polynomial time. For the case where $G_F$ is a directed acyclic graph, we gave a simple algorithm for counting the number of spanning arborescences. This algorithm formed the basis of a rejection sampling scheme that samples solutions to a PPM instance uniformly at random.

Using simulations, we showed that the number of solutions increases with increasing number *n* of mutations but decreases with increasing number *m* of samples. In addition, we showed that the median recall of all solutions increases with increasing *m* but decreases with increasing *n*. We showed how constraints from single-cell and long-read sequencing reduce the number of solutions. Finally, we showed that current MCMC methods fail to sample uniformly from the solution space. This is problematic as it leads to biases that propagate to downstream analyses.

There are a couple of avenues for future research. First, our hardness proof uses a reduction from SubsetSum, which has a pseudo-polynomial time algorithm. Recognizing that in practice the frequency matrix is composed of fractional values with small denominators (corresponding to the sequencing coverage), it will be interesting to study whether a similar pseudo-polynomial time algorithm may be devised for the PPM problem. Second, while the rejection sampling algorithm achieves uniformity, it does not scale to practical problem instance sizes. Further research is needed to develop sampling algorithms that achieve near-uniformity and have reasonable running time for practical problem instances. Third, just as single-cell sequencing and long-read sequencing impose constraints on the solution space of PPM, it will be worthwhile to include additional prior knowledge to further constrain the solution space (such as the use of constraints on migration for metastatic cancers [33, 35]). Finally, the PPM problem and the simulations in this paper assumed error-free data. Further research is needed to study the effect of sequencing, sampling and mapping errors. It is to be expected that the problem of non-uniqueness is further exacerbated with additional sources of uncertainty.

## Supplementary information

**Additional file 1. Supplementary Text: Implications of non-uniqueness in phylogenetic deconvolution ofbulk DNA samples of tumors**

## Data Availability

An implementation of the rejection sampling algorithm is available on https://github.com/elkebir-group/OncoLib. The data and scripts to generate the results are available on https://github.com/elkebir-group/PPM-NonUniq.
