# Supplementary material for: Implications of non-uniqueness in phylogenetic deconvolution of bulk DNA samples of tumors
Source: Algorithms Mol Biol. 2019 Sep 3;14:19. doi: 10.1186/s13015-019-0155-6 (PMC6719395; doi:10.1186/s13015-019-0155-6)
Supplement: Supplementary file 1 — Additional file 1. Supplementary Text: Implications of non-uniqueness in phylogenetic deconvolution ofbulk DNA samples of tumors [file 13015_2019_155_MOESM1_ESM.pdf]

RESEARCH

Additional file 1: Supplementary Text: Implications of Non-uniqueness in Phylogenetic Deconvolution of Bulk DNA Samples of Tumors

Yuanyuan Qi[1], Dikshant Pradhan[2] and Mohammed El-Kebir[1*]

Appendix A: Omitted Proofs

We start by defining #MONO-1-IN-3SAT.

Problem 1 (#MONO-1-IN-3SAT [1]) Given a Boolean formula $\phi = \bigwedge_{j=1}^{q}(y_{j,1} \vee y_{j,2} \vee y_{j,3})$ in 3-conjunctive normal form (3-CNF) with p variables $\{x_1, \ldots, x_p\}$ and q clauses where each clause has exactly three positive or three negative literals, find a truth assignment $\theta : [p] \to \{0, 1\}$ that satisfies each clause of ϕ with exactly one true literal.

Ref. [1] shows #P-completeness of #MONO-1-IN-3SAT. Here, we show #P-completeness of #SUBSETSUM by giving a parsimonious polynomial-time reduction from #MONO-1-IN-3SAT. We note that the same literal may appear multiple times in a clause. However, we may assume without loss of generality that for any $i \in [p]$, variable x_i appears in at least one clause of ϕ. To see why this is the case, observe that such unconstrained variables can be set either true or false, thus each resulting in a multiplicative factor of two on the number of solutions.

(Main Text) Lemma 12 There exists a parsimonious reduction from #MONO-1-IN-3SAT to #SUBSETSUM.

Proof Let ϕ be a Boolean formula in 3-CNF with p variables $\{x_1, \ldots, x_p\}$ and q clauses where each clause has exactly three positive or three negative literals.

Given ϕ, we construct a $2p \times (p + q)$ matrix $Q = [NM]$, whose first p columns correspond to matrix $N = [n_{i,j}]$ and remaning q columns correspond to matrix $M = [m_{i,j}]$, each having $2p$ rows. The idea is that each row $i \in [2p]$ of Q uniquely corresponds to a literal, i.e. if i is odd it corresponds to the positive literal $x_{(i+1)/2}$ and if i is even it corresponds to the negative literal $\neg x_{i/2}$. The entries $n_{i,j}$ of matrix N are defined as

$$n_{i,k} = \begin{cases} 1, & \lfloor \frac{i+1}{2} \rfloor = k, \\ 0, & \text{otherwise.} \end{cases}$$

To define matrix M, we introduce the function $g(j, y)$ which counts the number of occurrences of literal y in clause j of ϕ. Using the function g, we define matrix $M = [m_{i,j}]$ as

$$m_{i,j} = \begin{cases} g(j, x_{(i+1)/2}), & \text{if } i \text{ is odd}, \\ g(j, \neg x_{i/2}), & \text{if } i \text{ is even}. \end{cases}$$

Observe that matrix M provides a lossless encoding for ϕ, and it is trivial to reconstruct ϕ from M. Moreover, observe that Q can be obtained in polynomial time from ϕ.

We obtain a SUBSETSUM instance from matrix $Q = [NM]$ as follows. First, observe that entries of Q are in the set $\{0, 1, 2, 3\}$. Thus, rows $\{1, 2, \ldots, 2p\}$ of $Q = [q_{k,i}]$ correspond to positive integers defined as $S =$

$$\phi = (x_1 \vee x_2 \vee x_3) \wedge (\neg x_2 \vee \neg x_3 \vee \neg x_4),$$

$$\theta = \{x_1 = false, x_2 = true, x_3 = false, x_4 = true\},$$

$$N = \begin{pmatrix} 1 & 0 & 0 & 0 \\ 1 & 0 & 0 & 0 \\ 0 & 1 & 0 & 0 \\ 0 & 1 & 0 & 0 \\ 0 & 0 & 1 & 0 \\ 0 & 0 & 1 & 0 \\ 0 & 0 & 0 & 1 \\ 0 & 0 & 0 & 1 \end{pmatrix}, M = \begin{pmatrix} 1 & 0 \\ 0 & 0 \\ 1 & 0 \\ 0 & 1 \\ 1 & 0 \\ 0 & 1 \\ 0 & 0 \\ 0 & 1 \end{pmatrix}, \qquad \begin{pmatrix} Q_2 \\ Q_3 \\ Q_6 \\ Q_7 \end{pmatrix} = \begin{pmatrix} 1 & 0 & 0 & 0 & 0 & 0 \\ 0 & 1 & 0 & 0 & 1 & 0 \\ 0 & 0 & 1 & 0 & 0 & 1 \\ 0 & 0 & 0 & 1 & 0 & 0 \end{pmatrix}.$$

Figure S1 An example of reduction from #MONO-1-IN-3SAT to #SUBSETSUM. We show a truth assignment θ of the variables in #-MONO-1-IN-3SAT problem instance ϕ. Observe that each clause in ϕ is satisfied by exactly one literal. Moreover, we show the rows of $Q = [NM]$ corresponding to θ.

*Correspondence: melkebir@illinois.edu
[1]Department of Computer Science, University of Illinois at Urbana-Champaign, Champaign, IL 61801, USA
Full list of author information is available at the end of the article

$\{s_1, s_2, \ldots, s_{2p}\}$, where

$$s_k = \sum_{i=1}^{p+q} q_{k,i} \cdot 4^{p+q-i}, \qquad \forall k \in [2p].$$

We define the target sum as

$$t = \sum_{i=1}^{p+q} 4^{p+q-i}.$$

Clearly, S and t can be obtained in polynomial time from ϕ. Fig. S1 shows an example of this reduction.

To prove correctness of this reduction, we start by showing that (S,t) is a valid #SubsetSum instance. By construction, each integer s_i is nonnegative. The remaining requirement is that for distinct rows $i \neq j$ it holds that $s_i \neq s_j$. Let $i,j \in [2k]$ such that $i \neq j$. The indices of the corresponding variables of these two literals are given by $\lfloor \frac{i+1}{2} \rfloor$ and $\lfloor \frac{j+1}{2} \rfloor$, respectively. We distinguish two cases. First, if $\lfloor \frac{i+1}{2} \rfloor \neq \lfloor \frac{j+1}{2} \rfloor$, then, by construction, $n_{i,\lfloor \frac{i+1}{2} \rfloor} \neq n_{j,\lfloor \frac{i+1}{2} \rfloor}$, i.e. the $\lfloor \frac{i+1}{2} \rfloor$-th digit of s_i in the base 4 representation of the numbers differs from s_j, and hence $s_i \neq s_j$. Second, if $\lfloor \frac{i+1}{2} \rfloor = \lfloor \frac{j+1}{2} \rfloor = k$, assume without loss of generality that $i = 2k-1$ and $j = 2k$. We have that $n_{i,l} = n_{j,l}$ for all $l \in [p]$. We claim that there is a clause $s \in [q]$ that contains at most one of $\{x_k, \neg x_k\}$. Suppose for a contradiction that no clause of ϕ contains at most one $\{x_k, \neg x_k\}$. If a clause of ϕ contains both x_k and $\neg x_k$ then it violates the monochromaticity property of ϕ. If no clause of ϕ contains either x_k or $\neg x_k$ then x_k is not a variable of ϕ. Thus, there exists a clause $l \in [p]$ that contains at most one of $\{x_k, \neg x_k\}$. As such, for this clause l we have $m_{i,l} \neq m_{i,l}$ and thus $s_i \neq s_j$. Hence, (S,t) is a valid #SubsetSum instance.

To prove that this reduction is parsimonious, we need to prove that the number of solutions (witnesses) is preserved. We prove this by establishing a bijection between the set of satisfying truth assignments of ϕ and the set of subsets of S that sum to the target sum d. Consider a satisfying assignment θ of ϕ. We construct the corresponding subset D of rows $\{1,\ldots,2p\}$ of Q as follows. For each variable x_k (where $k \in [p]$), we include s_{2k-1} in D if $\theta(x_k) = 1$ and include s_{2k} in D otherwise. Let $d = \sum_{i \in D} s_i$. By construction, only one of $\{s_{2k-1}, s_{2k}\}$ is included in D. As such, the k-th digit of d is 1, for all $k \in [p]$. Moreover, since exactly one literal of each clause is true, the i-th digit of d is 1 for each $i \in \{p+1,\ldots,p+q\}$. Therefore D is a subset of the rows of Q such that $\sum_{i \in D} s_i = t$.

Consider a subset D of S such that $\sum_{i \in D} s_i = t$. Since the values of each entry in Q are in the set $\{0,1,2,3\}$, each row $i \in D$ describes a quaternary number. We claim that adding the quaternary numbers corresponding to D will not introduce a carry. To see this, recall that $Q = [NM]$. For the k-th column of N (where $k \in [p]$), the entry $n_{i,k}$ in row i has value 1 if and only if $\lfloor \frac{i+1}{2} \rfloor = k$. This is only the case for $i = 2k-1$ or $i = 2k$, and hence only two entries in each column N are 1. For columns of M, the sum of the entries in j-th column is $\sum_{k \in [p]} [g(j,x_k) + g(j,\neg x_k)]$, i.e. the sum of the number of the occurrences of all literals in clause j. Since we only have three literals in each clause, this sum equals exactly 3. Therefore, no carry will happen in the addition under the quaternary representation, and since the k-th digit of t under the quaternary representation is 1 for $1 \leq k \leq p$, only one of $\{s_{2k-1}, s_{2k}\}$ is in D. We construct an assignment θ of ϕ, where we set $\theta(x_k) = 1$ if $s_{2k-1} \in D$ and set $\theta(x_k) = 0$ if $s_{2k} \in D$. Since for all $i \in \{p+1,\ldots,p+q\}$ the i-th digit of t is 1, only one literal of each clause is true. Hence, θ is a satisfying assignment.

Obviously, the construction of D given θ and the construction of θ given D are inverses of each other. Therefore, if we view the constructions as functions, they are bijections between the solution spaces. This proves that the reduction is parsimonious. □

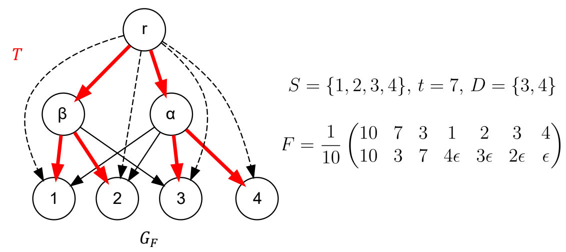

Figure S2 An example reduction from a #SubsetSum instance to #PPM. A solution to the SubsetSum problem instance (S,t) and its corresponding spanning arborescence T of ancestry graph G_F.

(Main Text) Lemma 13 There exists a parsimonious reduction from #SubsetSum to #PPM restricted to $m = 2$ samples.

Proof El-Kebir et al. [2] showed the NP-completeness of PPM even when $m = 2$ by reduction from SubsetSum. Here, we will show that this reduction is parsimonious by defining a bijection between the solution space of a SubsetSum instance and the solution space of the corresponding PPM instance. From this result

it will immediately follow that the number of solutions is preserved.

Let (S, t) be an instance of SubsetSum, where $S = \{s_1, s_2, \ldots, s_d\}$ is the set of numbers and t is the target sum. Suppose $s_1 < s_2 < \ldots < s_d$ without loss of generality. Let

$$F = \frac{1}{e}\begin{pmatrix} e & t & e-t & s_1 & \cdots & s_d \\ e & e-t & t & s_d\epsilon & \cdots & s_1\epsilon \end{pmatrix},$$

where $e = \sum_{s \in S} s$, and $0 < \epsilon < \frac{1}{e}$. Clearly, F can be obtained in polynomial time, and is a valid instance of PPM. Let $G_F = (V, E)$ be the ancestry graph for F, whose vertices $r, \alpha, \beta, v_1, v_2, \ldots, v_d$ correspond to the columns of F.

Now consider a subset $D \subseteq S$ whose sum $\sum_{s_i \in D} s_i$ equals the target value t. Given D, we construct a spanning arborescence $T(D) = (V, E')$ of G_F, where

$$E' = \{(\alpha, v_i) \mid s_i \in D\} \cup \{(\beta, v_i) \mid s_i \notin D\} \cup \{(r, \alpha), (r, \beta)\}.$$

For any i, vertex v_i is connected to either α or β, and α and β are both connected to r. Therefore, each vertex is reachable from r. Since $|E'| = |D| + |S - D| + 2 = d + 2$ and $|V| = d + 3$, T is an arborescence over V. Now we need to show that T is a spanning arborescence of G_F and it suffices to show $E' \subseteq E$. First, $(r, \alpha), (r, \beta)$ are obviously included in E. Since $\sum_{s_i \in D} s_i = t$ and $s_i > 0$ for all $i \in [d]$, it holds that $s_i \leq t$. Thus, $f_{1,v_i} \leq f_{1,\alpha}$ if $s_i \in D$. Wit a similar argument, we can show that $f_{1,v_i} \leq f_{1,\beta}$ if $s_i \notin D$. Since $0 < t < e$ and e, t are integers, we have that $t \geq 1, e - t \geq 1$. Since $f_{2,v_i} = s_i\epsilon < s_i/e < 1$, we have $\{(\alpha, v_i) | s_i \in D\} \cup \{(\beta, v_i) | s_i \notin D\} \subseteq E$, and thus $E' \subseteq E$. To show that $T(D)$ is a solution to the PPM, we need to show that $T(D)$ also satisfies (SC). Since $T(D)$ has only three non-leaf vertices $\{r, \alpha, \beta\}$, it suffices to verify (SC) at these three vertices. Obviously, (SC) holds for r. Since

$$f_{1,\alpha} = \frac{t}{e} = \frac{\sum_{s_i \in D} s_i}{e} = \sum_{s_i \in D} f_{1,v_i}$$

and

$$f_{2,\alpha} \geq \frac{1}{e} > \frac{\sum_{i \in [d]} s_i}{e}\epsilon = \sum_{i \in [d]} f_{2,v_i} > \sum_{s_i \in D} f_{2,v_i},$$

(SC) holds for α. Similarly, we can show that (SC) holds for β. Therefore, $T(D)$ is a valid solution to the instance F of PPM.

Consider a spanning arborescence $T = (V, E')$ of G_F that satisfies (SC). The edge set $E' \subseteq E$ satisfies the following:

(i) $(r, \alpha), (r, \beta) \in E'$.
Since $f_{2,\alpha} \geq \frac{1}{e} > \frac{\sum_{i \in [d]} s_i}{e}\epsilon = \sum_{i \in [d]} f_{2,v_i} > f_{2,v_i}$, it holds that $(v_i, \alpha) \notin E$ for any $i \in [d]$. Similarly, $(v_i, \beta) \notin E$ for any $i \in [d]$. Therefore, α, β can only be reachable from r.

(ii) $(r, v_i) \notin E'$ for any $i \in [d]$.
Since $(r, \alpha), (r, \beta) \in E'$ and $F_{1,\alpha} + F_{1,\beta} = F_{1,r}$, adding any (r, v_i) will violate (SC) at r.

(iii) For any distinct $i, j \in [d]$, it holds that $(v_i, v_j) \notin E$.
Suppose $i > j$, then $f_{1,v_i} > f_{1,v_j}$ and $f_{2,v_i} < f_{2,v_j}$. Therefore, $(v_i, v_j) \notin E$.

(iv) Either (α, v_i) or (β, v_i) is in E' for any $i \in [d]$.
In (ii) and (iii), we have shown that v_i is not a child of r, or a child of any other v_j (where $j \neq i$). Therefore, v_i can only be reached from r through α or β.

Fig. S2 shows an example. Let $D(T) = \{s_i \mid (\alpha, v_i) \in E'\}$. By (SC), we have that $\sum_{s_i \in D} s_i \leq t$ and $\sum_{s_i \notin D(T)} s_i \leq e - t$. Since $\sum_{s_i \in D(T)} s_i + \sum_{s_i \notin D(T)} s_i = \sum_{s_i \in S} s_i = e$, both inequalities are tight, i.e. $\sum_{s_i \in D(T)} s_i = t$ and $\sum_{s_i \notin D(T)} s_i = e - t$. Therefore, $D(T)$ is a solution to SubsetSum.

The construction of T given D and the construction of D given T are inverses of each other. If we view the constructions as functions, they are bijections between the solution spaces. This in turn shows that the number of solutions (witnesses) is preserved. Obviously, the reduction can be performed in polynomial time. Therefore, this reduction is parsimonious. □

Appendix B: Supplementary Results

We have the following additional figures and tables in the supplement.

- Fig. S3 illustrates how an ancestry graph is derived from a frequency matrix.
- Fig. S4 shows the solution space of lung cancer patient CRUK0012 with $m = 2$ samples and $n = 5$ mutation clusters.
- Fig. S5 shows the six solutions of instance #81 with $n = 7$ mutations and $m = 5$ samples.
- Fig. S6 illustrates the distribution of samples drawn by PhyloWGS for all $n = 7$ instances.
- Fig. S7 illustrates the distribution of samples drawn by Canopy for all $n = 7$ instances.
- Fig. S8 illustrates the distribution of samples drawn by rejection sampling for all $n = 7$ instances.
- Tables S1-S2 list the real data results obtained from a lung cancer cohort [3].
- Table S3 lists the parameters and results of all simulation instances where $n = 3$.
- Table S4 lists the parameters and results of all simulation instances where $n = 5$.

- Table S5 lists the parameters and results of all simulation instances where $n = 7$.
- Table S6 lists the parameters and results of all simulation instances where $n = 9$.
- Table S7 lists the parameters and results of all simulation instances where $n = 11$.
- Table S8 lists the parameters and results of all simulation instances where $n = 13$.
- Table S9 lists the parameters and results of rejection sampling over all $n = 7$ simulation instances.

Table S1 Non-uniqueness of solutions in a multi-region lung cancer cohort of 100 patients [3]. Table shows the patient identifier, the number n of mutation clusters, the number m of samples, the number of solutions in the solution space when removing no samples, one sample and two samples, and finally the number of solutions reported in Ref. [3]. Patients where our enumeration algorithm found no solutions are indicated with *. Patients where no solutions were reported in Ref. [3] are indicated with **.

patient	clusters n	samples m	0 removed samples	1 removed sample	2 removed samples	solutions reported in [3]
CRUK0001	7	3	0*	24	3936	1
CRUK0002	8	3	6	84	53248	1
CRUK0003	8	5	86	238	1680	1
CRUK0004	7	4	30	102	735	2
CRUK0005	6	4	2	10	48	2
CRUK0006	3	2	1	4	N/A	1
CRUK0007	3	2	1	4	N/A	1
CRUK0008	5	2	4	60	N/A	1
CRUK0009	8	4	4	14	1920	1
CRUK0010	4	2	2	12	N/A	1
CRUK0011	8	3	27	768	49152	3
CRUK0012	5	2	6	135	N/A	2
CRUK0013	9	5	480	2343	2343	8
CRUK0014	3	2	1	4	N/A	1
CRUK0015	4	2	2	12	N/A	1
CRUK0016	8	2	0*	3941	N/A	1
CRUK0017	8	4	0*	28	336	1
CRUK0018	8	4	4	56	1920	1
CRUK0019	3	2	1	4	N/A	1
CRUK0020	4	2	2	27	N/A	1
CRUK0021	4	2	2	16	N/A	1
CRUK0022	5	2	3	90	N/A	2
CRUK0023	10	4	54	1680	29400	2
CRUK0024	8	4	12	192	1280	1
CRUK0025	7	3	18	84	4116	2
CRUK0026	3	2	1	4	N/A	1
CRUK0027	5	2	4	60	N/A	1
CRUK0028	5	2	4	60	N/A	2
CRUK0029	9	6	108	1224	11920	1
CRUK0030	4	3	2	3	16	1
CRUK0031	7	3	21	140	3887	2
CRUK0032	13	4	636	75790	19649968	1
CRUK0033	2	2	1	1	N/A	1
CRUK0034	7	3	6	420	9702	1
CRUK0035	5	4	1	8	45	1
CRUK0036	7	4	2	12	140	1
CRUK0037	10	5	828	14774	75660	17
CRUK0038	4	2	2	12	N/A	2
CRUK0039	8	3	3	288	36864	1
CRUK0040	2	2	2	2	N/A	1
CRUK0041	3	4	1	4	4	1
CRUK0043	2	2	1	2	N/A	1
CRUK0044	3	2	1	4	N/A	1
CRUK0045	3	2	1	4	N/A	1
CRUK0046	5	4	5	14	19	2
CRUK0047	3	2	1	4	N/A	1
CRUK0048	4	3	4	4	15	1
CRUK0049	6	2	10	294	N/A	4
CRUK0050	3	5	2	2	2	1

Table S2 Non-uniqueness of solutions in a multi-region lung cancer cohort of 100 patients [3]. Table shows the patient identifier, the number n of mutation clusters, the number m of samples, the number of solutions in the solution space when removing no samples, one sample and two samples, and finally the number of solutions reported in Ref. [3]. Patients where our enumeration algorithm found no solutions are indicated with *. Patients where no solutions were reported in Ref. [3] are indicated with **.

patient	clusters n	samples m	0 removed samples	1 removed sample	2 removed samples	solutions reported in [3]
CRUK0051	5	3	2	12	105	1
CRUK0052	5	3	2	8	90	1
CRUK0054	3	2	1	2	N/A	1
CRUK0055	2	1	1	N/A	N/A	0**
CRUK0056	4	3	1	3	18	1
CRUK0057	4	2	1	18	N/A	1
CRUK0061	2	2	1	1	N/A	1
CRUK0062	15	7	160	19500	289500	1
CRUK0063	8	5	65	847	1330	2
CRUK0064	5	2	2	72	N/A	1
CRUK0065	14	6	3280	8710	173712	1
CRUK0066	8	4	5	28	672	1
CRUK0067	5	2	5	90	N/A	2
CRUK0068	10	4	328	1485	20160	3
CRUK0069	12	5	1464	565216	3411072	1
CRUK0070	10	5	56	2136	160800	2
CRUK0071	10	6	180	540	7200	1
CRUK0072	6	3	3	25	552	1
CRUK0073	3	2	1	4	N/A	1
CRUK0074	3	2	1	4	N/A	1
CRUK0075	3	2	1	4	N/A	1
CRUK0076	9	4	157	504	17010	4
CRUK0077	7	4	35	328	1155	2
CRUK0078	6	3	1	40	552	1
CRUK0079	6	4	8	25	120	1
CRUK0080	4	4	1	1	6	1
CRUK0081	3	2	1	4	N/A	1
CRUK0082	6	5	2	85	137	1
CRUK0083	8	4	10	56	1152	1
CRUK0084	6	4	8	40	332	2
CRUK0085	10	4	48	8160	750000	1
CRUK0086	3	3	1	2	3	1
CRUK0087	3	3	1	2	2	1
CRUK0088	3	2	2	5	N/A	1
CRUK0090	2	2	1	2	N/A	1
CRUK0094	6	4	14	122	224	2
CRUK0095	4	3	6	6	17	2
CRUK0096	5	7	1	2	15	1
CRUK0097	4	2	2	12	N/A	1
CRUK0098	4	3	2	2	16	1
CRUK0099	5	4	3	4	15	2
CRUK0100	8	3	60	315	26880	3

Table S3 Result for $n = 3$ instances. From left to right, we list the instance identifier, the number m of samples, the number of solutions, the number of spanning arborescences in the ancestry graph of the instance, the ratio between the solutions and spanning arborescences and the median edge recall.

#	samples m	solutions	spanning arborescences	ratio	median recall
2	1	2	2	1.000000	0.750
	2	1	2	0.500000	1.000
	5	1	2	0.500000	1.000
	10	1	2	0.500000	1.000
8	1	2	2	1.000000	0.750
	2	2	2	1.000000	0.750
	5	1	1	1.000000	1.000
	10	1	1	1.000000	1.000
12	1	2	2	1.000000	0.750
	2	2	2	1.000000	0.750
	5	1	1	1.000000	1.000
	10	1	1	1.000000	1.000
15	1	2	2	1.000000	0.750
	2	2	2	1.000000	0.750
	5	1	1	1.000000	1.000
	10	1	1	1.000000	1.000
30	1	2	2	1.000000	0.750
	2	2	2	1.000000	0.750
	5	1	1	1.000000	1.000
	10	1	1	1.000000	1.000
39	1	2	2	1.000000	0.750
	2	2	2	1.000000	0.750
	5	1	1	1.000000	1.000
	10	1	1	1.000000	1.000
50	1	2	2	1.000000	0.750
	2	2	2	1.000000	0.750
	5	1	1	1.000000	1.000
	10	1	1	1.000000	1.000
104	1	2	2	1.000000	0.750
	2	2	2	1.000000	0.750
	5	1	1	1.000000	1.000
	10	1	1	1.000000	1.000
119	1	2	2	1.000000	0.750
	2	2	2	1.000000	0.750
	5	1	1	1.000000	1.000
	10	1	1	1.000000	1.000
129	1	2	2	1.000000	0.750
	2	2	2	1.000000	0.750
	5	1	1	1.000000	1.000
	10	1	1	1.000000	1.000

Table S4 **Result for $n = 5$ instances**. From left to right, we list the instance identifier, the number m of samples, the number of solutions, the number of spanning arborescences in the ancestry graph of the instance, the ratio between the solutions and spanning arborescences and the median edge recall.

#	samples m	solutions	spanning arborescences	ratio	median recall
3	1	15	24	0.625000	0.500
	2	6	12	0.500000	0.750
	5	4	6	0.666667	0.625
	10	1	2	0.500000	1.000
5	1	16	24	0.666667	0.500
	2	5	6	0.833333	0.750
	5	5	6	0.833333	0.750
	10	2	2	1.000000	0.875
9	1	21	24	0.875000	0.500
	2	3	6	0.500000	0.750
	5	9	24	0.375000	0.500
	10	2	6	0.333333	0.875
18	1	21	24	0.875000	0.500
	2	5	6	0.833333	0.750
	5	6	8	0.750000	0.750
	10	2	3	0.666667	0.875
37	1	24	24	1.000000	0.500
	2	6	6	1.000000	0.750
	5	6	6	1.000000	0.750
	10	2	2	1.000000	0.875
45	1	12	24	0.500000	0.625
	2	3	16	0.187500	0.750
	5	5	24	0.208333	0.750
	10	2	18	0.111111	0.875
62	1	18	24	0.750000	0.500
	2	5	6	0.833333	0.750
	5	6	6	1.000000	0.750
	10	2	2	1.000000	0.875
66	1	11	24	0.458333	0.500
	2	4	12	0.333333	0.750
	5	2	6	0.333333	0.875
	10	2	6	0.333333	0.875
69	1	22	24	0.916667	0.500
	2	4	6	0.666667	0.625
	5	7	8	0.875000	0.750
	10	2	3	0.666667	0.875
71	1	11	24	0.458333	0.750
	2	4	18	0.222222	0.750
	5	2	24	0.083333	0.875
	10	1	18	0.055556	1.000

Table S5 Result for $n = 7$ instances. From left to right, we list the instance identifier, the number m of samples, the number of solutions, the number of spanning arborescences in the ancestry graph of the instance, the ratio between the solutions and spanning arborescences and the median edge recall.

#	samples m	solutions	spanning arborescences	ratio	median recall
7	1	432	720	0.600000	0.500
	2	94	120	0.783333	0.500
	5	24	60	0.400000	0.667
	10	6	24	0.250000	0.833
10	1	28	720	0.038889	0.667
	2	17	720	0.023611	0.667
	5	4	144	0.027778	0.833
	10	3	144	0.020833	0.833
12	1	315	720	0.437500	0.333
	2	43	120	0.358333	0.500
	5	12	80	0.150000	0.750
	10	6	48	0.125000	0.833
23	1	79	720	0.109722	0.500
	2	18	360	0.050000	0.667
	5	10	180	0.055556	0.750
	10	3	90	0.033333	0.833
30	1	293	720	0.406944	0.500
	2	70	120	0.583333	0.667
	5	22	24	0.916667	0.667
	10	6	6	1.000000	0.833
43	1	618	720	0.858333	0.333
	2	54	720	0.075000	0.500
	5	21	360	0.058333	0.667
	10	6	216	0.027778	0.833
49	1	398	720	0.552778	0.333
	2	37	270	0.137037	0.500
	5	2	24	0.083333	0.917
	10	1	24	0.041667	1.000
61	1	328	720	0.455556	0.500
	2	106	240	0.441667	0.500
	5	19	30	0.633333	0.667
	10	3	8	0.375000	0.833
66	1	101	720	0.140278	0.500
	2	14	240	0.058333	0.667
	5	6	120	0.050000	0.833
	10	2	48	0.041667	0.917
81	1	297	720	0.412500	0.500
	2	50	240	0.208333	0.667
	5	6	48	0.125000	0.833
	10	2	24	0.083333	0.917

Table S6 Result for $n = 9$ instances. From left to right, we list the instance identifier, the number m of samples, the number of solutions, the number of spanning arborescences in the ancestry graph of the instance, the ratio between the solutions and spanning arborescences and the median edge recall.

#	samples m	solutions	spanning arborescences	ratio	median recall
0	1	1472	40320	0.036508	0.500
	2	36	1920	0.018750	0.750
	5	7	360	0.019444	0.875
	10	5	360	0.013889	0.875
5	1	10445	40320	0.259053	0.375
	2	2200	5040	0.436508	0.500
	5	4	16	0.250000	0.875
	10	3	12	0.250000	0.875
18	1	6180	40320	0.153274	0.500
	2	1450	5040	0.287698	0.500
	5	13	60	0.216667	0.750
	10	9	48	0.187500	0.750
24	1	4776	40320	0.118452	0.375
	2	522	10080	0.051786	0.500
	5	36	1440	0.025000	0.625
	10	12	960	0.012500	0.750
27	1	3755	40320	0.093130	0.500
	2	382	7560	0.050529	0.625
	5	16	864	0.018519	0.750
	10	6	360	0.016667	0.875
31	1	8183	40320	0.202951	0.375
	2	600	13440	0.044643	0.500
	5	19	288	0.065972	0.750
	10	6	180	0.033333	0.875
32	1	14760	40320	0.366071	0.375
	2	1196	3360	0.355952	0.500
	5	56	720	0.077778	0.625
	10	18	120	0.150000	0.688
48	1	9436	40320	0.234028	0.375
	2	1906	10080	0.189087	0.375
	5	36	240	0.150000	0.625
	10	9	48	0.187500	0.750
56	1	10122	40320	0.251042	0.375
	2	1234	2016	0.612103	0.500
	5	66	120	0.550000	0.750
	10	6	16	0.375000	0.875
70	1	22151	40320	0.549380	0.375
	2	3364	10080	0.333730	0.375
	5	13	80	0.162500	0.750
	10	7	48	0.145833	0.875

Table S7 Result for $n = 11$ instances. From left to right, we list the instance identifier, the number m of samples, the number of solutions, the number of spanning arborescences in the ancestry graph of the instance, the ratio between the solutions and spanning arborescences and the median edge recall.

#	samples m	solutions	spanning arborescences	ratio	median recall
24	1	74138	3628800	0.020430	0.400
	2	11301	1451520	0.007786	0.400
	5	4	2160	0.001852	0.900
	10	2	1296	0.001543	0.950
27	1	211022	3628800	0.058152	0.400
	2	9456	544320	0.017372	0.500
	5	46	12096	0.003803	0.700
	10	3	2592	0.001157	0.900
35	1	13338	3628800	0.003676	0.500
	2	3350	3628800	0.000923	0.600
	5	10	108000	0.000093	0.850
	10	3	72000	0.000042	0.900
69	1	224451	3628800	0.061853	0.400
	2	3898	120960	0.032226	0.600
	5	15	3840	0.003906	0.800
	10	5	1920	0.002604	0.900
83	1	129706	3628800	0.035743	0.400
	2	936	414720	0.002257	0.600
	5	104	40320	0.002579	0.600
	10	4	4320	0.000926	0.900
89	1	4249	3628800	0.001171	0.500
	2	547	1814400	0.000301	0.700
	5	4	15120	0.000265	0.900
	10	3	12960	0.000231	0.900
109	1	546559	3628800	0.150617	0.400
	2	78547	362880	0.216454	0.500
	5	48	480	0.100000	0.800
	10	7	64	0.109375	0.900
115	1	288866	3628800	0.079604	0.300
	2	6428	241920	0.026571	0.400
	5	6	192	0.031250	0.900
	10	6	512	0.011719	0.900
129	1	522216	3628800	0.143909	0.400
	2	103994	725760	0.143290	0.400
	5	60	640	0.093750	0.750
	10	12	96	0.125000	0.800
139	1	729024	3628800	0.200899	0.400
	2	84747	725760	0.116770	0.400
	5	10	432	0.023148	0.850
	10	8	216	0.037037	0.850

Table S8 Result for $n = 13$ instances. From left to right, we list the instance identifier, the number m of samples, the number of solutions, the number of spanning arborescences in the ancestry graph of the instance, the ratio between the solutions and spanning arborescences and the median edge recall.

#	samples m	solutions	spanning arborescences	ratio	median recall
3	1	9863339	479001600	0.020591	0.250
	2	409393	47900160	0.008547	0.417
	5	252	194400	0.001296	0.667
	10	4	11520	0.000347	0.917
12	1	1118667	479001600	0.002335	0.250
	2	14892	19958400	0.000746	0.583
	5	16	138240	0.000116	0.833
	10	6	40320	0.000149	0.917
15	1	867056	479001600	0.001810	0.417
	2	26834	114048000	0.000235	0.500
	5	42	2419200	0.000017	0.750
	10	4	829440	0.000005	0.917
19	1	7318619	479001600	0.015279	0.333
	2	120419	65318400	0.001844	0.500
	5	60	97200	0.000617	0.750
	10	6	38880	0.000154	0.917
25	1	78781	479001600	0.000164	0.500
	2	2488	119750400	0.000021	0.667
	5	44	1244160	0.000035	0.750
	10	3	345600	0.000009	0.917
40	1	9300931	479001600	0.019417	0.250
	2	436744	47900160	0.009118	0.333
	5	352	40320	0.008730	0.667
	10	12	4800	0.002500	0.833
43	1	33809749	479001600	0.070584	NA
	2	575588	29030400	0.019827	0.333
	5	152	69120	0.002199	0.667
	10	7	5760	0.001215	0.917
45	1	28053	479001600	0.000059	0.583
	2	1592	108864000	0.000015	0.667
	5	20	2646000	0.000008	0.833
	10	2	1620000	0.000001	0.958
56	1	23086684	479001600	0.048198	NA
	2	2235187	79833600	0.027998	0.333
	5	280	57600	0.004861	0.667
	10	12	3840	0.003125	0.833
84	1	27236653	479001600	0.056861	NA
	2	8623319	479001600	0.018003	0.333
	5	156	3600	0.043333	0.667
	10	12	768	0.015625	0.833

Table S9 Rejection sampling results for $n = 7$ instances. From left to right, we list the instance identifier, the number m of samples, the number of solutions (satisfying (SC)), the number of spanning arborescences in the ancestry graph of the instance, the ratio between the solutions and spanning trees, the total number of samples (trials) used by the rejection sampling algorithm, the fraction of accepted samples (successful trials). Observe that 'success ratio' ≈ 'solution ratio'.

#	samples m	solutions	spanning arborescences	solution ratio	trials	success ratio
7	1	432	720	0.600	16585	0.603
	2	94	120	0.783	12753	0.784
	5	24	60	0.400	24821	0.403
	10	6	24	0.250	40859	0.245
10	1	28	720	0.039	256090	0.039
	2	17	720	0.024	419637	0.024
	5	4	144	0.028	358360	0.028
	10	3	144	0.021	481517	0.021
12	1	315	720	0.438	23109	0.433
	2	43	120	0.358	28009	0.357
	5	12	80	0.150	67803	0.147
	10	6	48	0.125	78530	0.127
23	1	79	720	0.110	90828	0.110
	2	18	360	0.050	197369	0.051
	5	10	180	0.056	180518	0.055
	10	3	90	0.033	300223	0.033
30	1	293	720	0.407	24665	0.405
	2	70	120	0.583	17204	0.581
	5	22	24	0.917	10942	0.914
	10	6	6	1.000	10000	1.000
43	1	618	720	0.858	11606	0.862
	2	54	720	0.075	132441	0.076
	5	21	360	0.058	169685	0.059
	10	6	216	0.028	354898	0.028
49	1	398	720	0.553	18115	0.552
	2	37	270	0.137	73073	0.137
	5	2	24	0.083	120731	0.083
	10	1	24	0.042	239816	0.042
61	1	328	720	0.456	21939	0.456
	2	106	240	0.442	22626	0.442
	5	19	30	0.633	15896	0.629
	10	3	8	0.375	26864	0.372
66	1	101	720	0.140	71260	0.140
	2	14	240	0.058	171753	0.058
	5	6	120	0.050	199703	0.050
	10	2	48	0.042	239576	0.042
81	1	297	720	0.412	24528	0.408
	2	50	240	0.208	49137	0.204
	5	6	48	0.125	79423	0.126
	10	2	24	0.083	120821	0.083

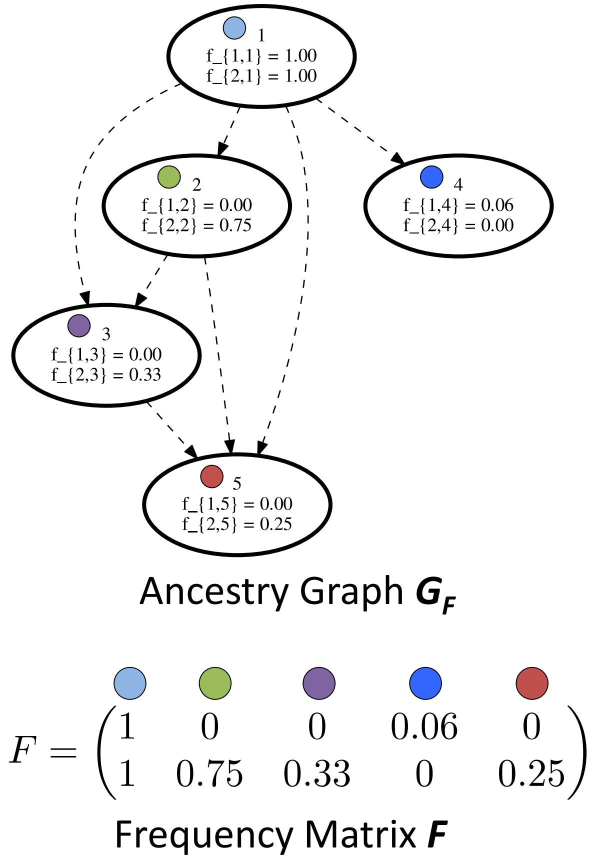

Figure S3 Example ancestry graph. Frequency matrix F corresponds to a simulated $n = 5$ instance (#9) and has $m = 2$ samples. The corresponding ancestry graph G_F illustrates the potential parental relationships.

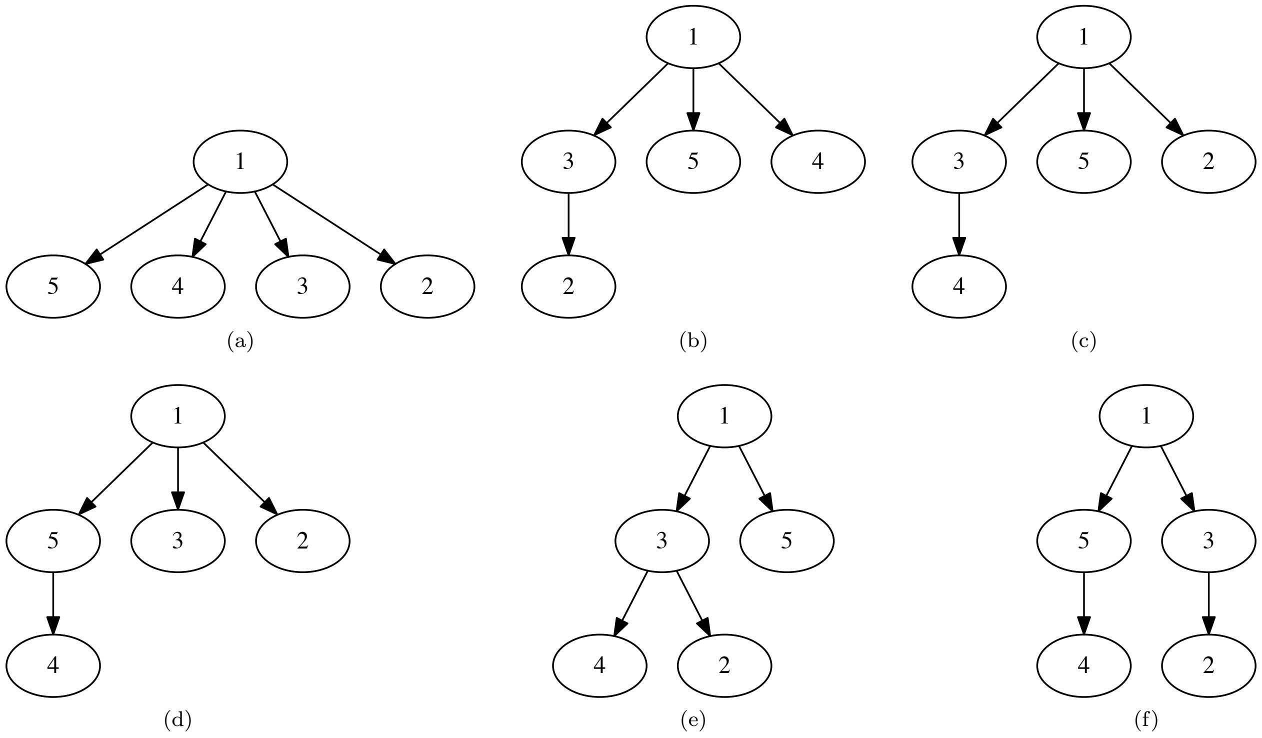

Figure S4 Patient CRUK0012 with $m = 2$ samples and $n = 5$ mutation clusters has six possible trees. Jamal-Hanjani et al. [3] reported two trees, which correspond to (e) and (f). The solution space contains four more trees that were not reported in [3].

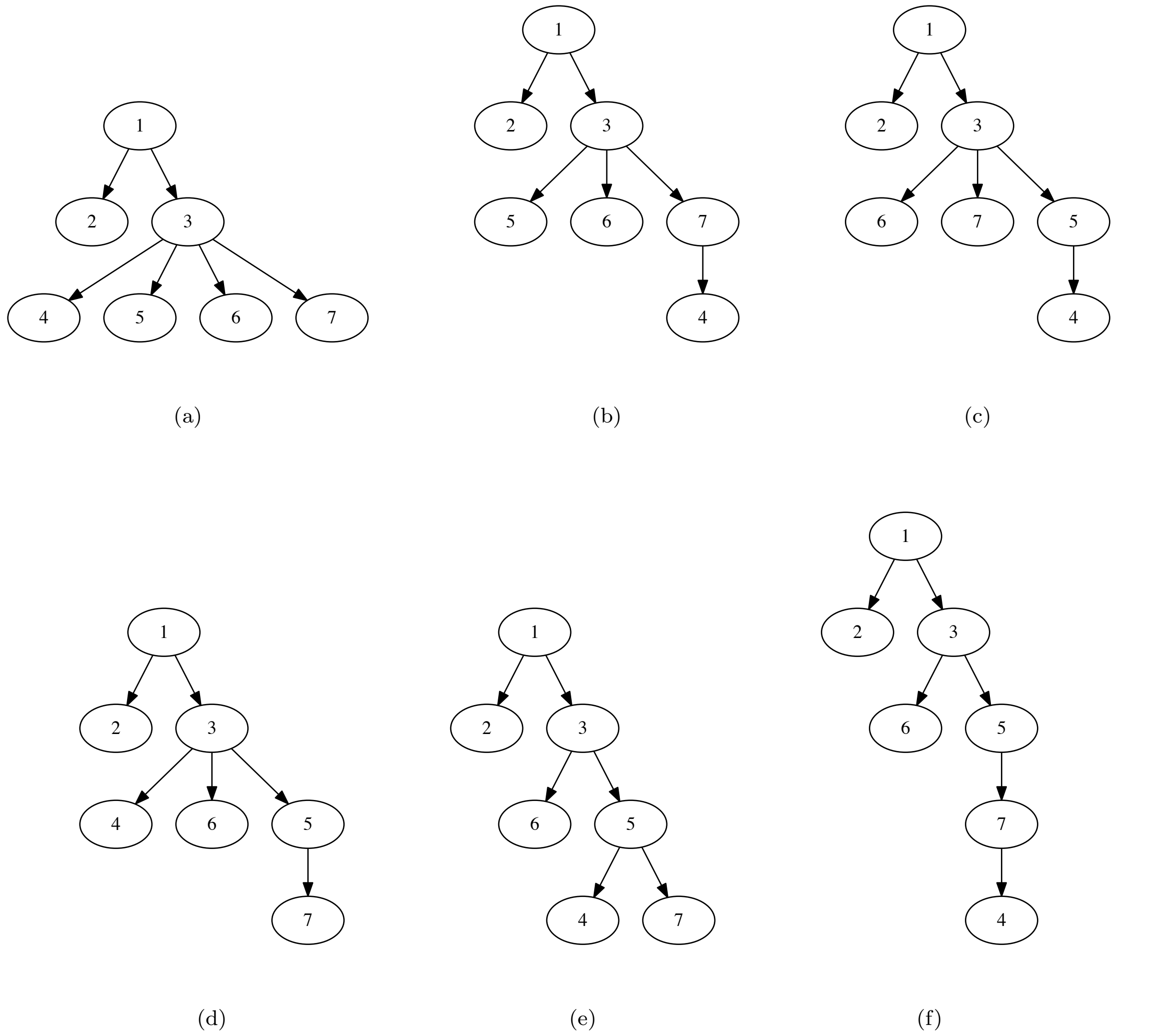

Figure S5 Instance #81 with $n = 7$ mutations and $m = 5$ samples has six solutions. Solution (A) is the true solution. All 7500 samples generated by PhyloWGS correspond to (F). Canopy generated a total of 387 samples corresponding to three different trees. Two out of the three trees were incorrect (307 samples), the remaining 80 samples correspond to (A). Our rejection sampling procedure generated 10000 samples corresponding to each of the six trees in roughly equal proportions.

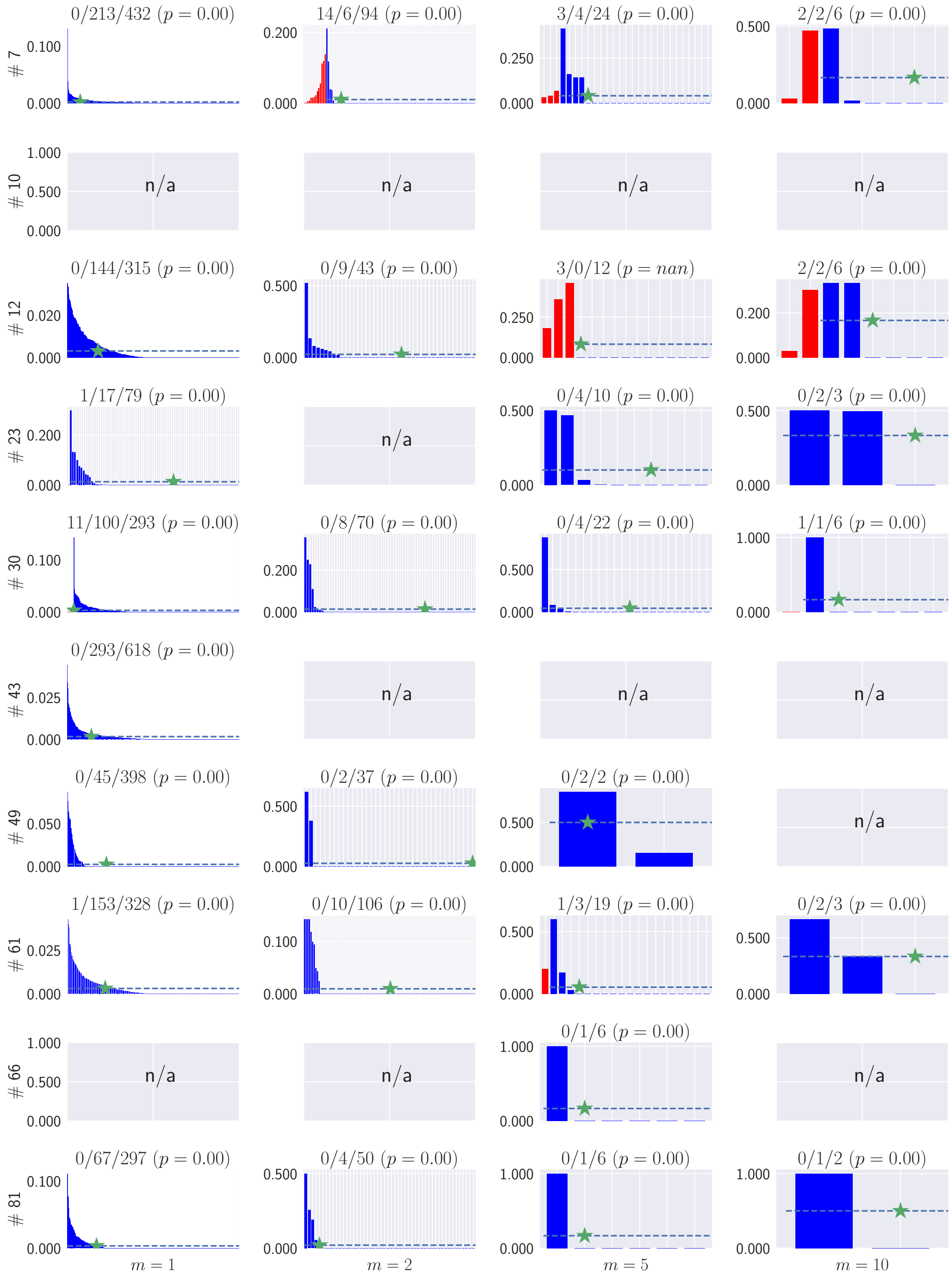

Figure S6 PhyloWGS results. Each plot shows the relative frequency of correct solutions (satisfying (SC)) output by PhyloWGS (blue bars), with the simulated solution indicated by '$\star$'. Red bars correspond to incorrect solutions (violating (SC)). Dashed line indicates the expected relative frequency in the case of uniformity. The title of each plot lists the number of incorrect solutions, the number of recovered correct solutions, the total number of correct solutions and the p-value of the chi-squared test of uniformity. PhyloWGS did not generate any trees without clustered mutations for the instances marked by 'n/a'.

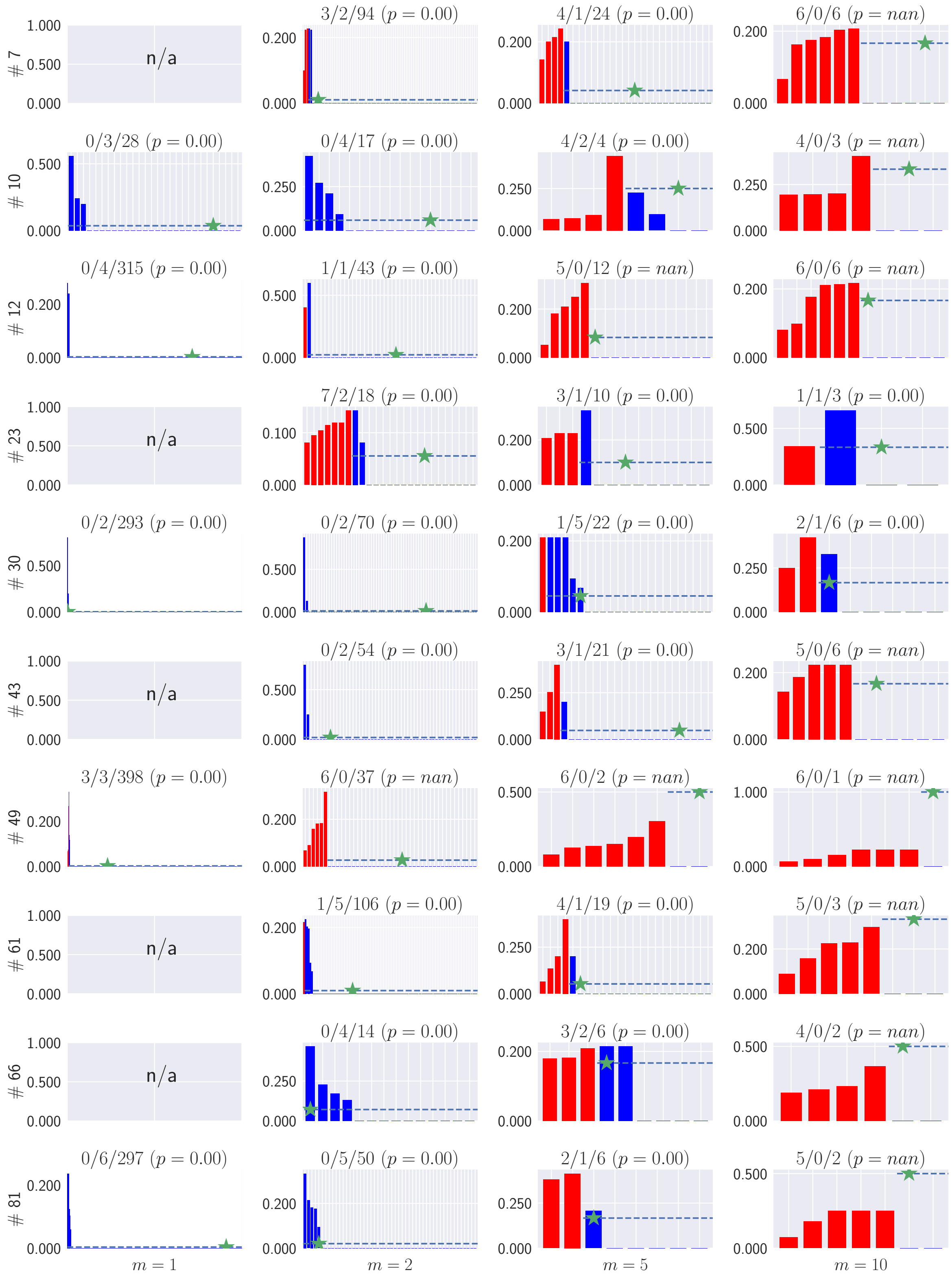

Figure S7 Canopy results. Each plot shows the relative frequency of correct solutions (satisfying (SC)) output by Canopy (blue bars), with the simulated solution indicated by '⋆'. Red bars correspond to incorrect solutions (violating (SC)). Dashed line indicates the expected relative frequency in the case of uniformity. The title of each plot lists the number of incorrect solutions, the number of recovered correct solutions, the total number of correct solutions and the p-value of the chi-squared test of uniformity. Canopy did not generate any trees without clustered mutations for the instances marked by 'n/a'.

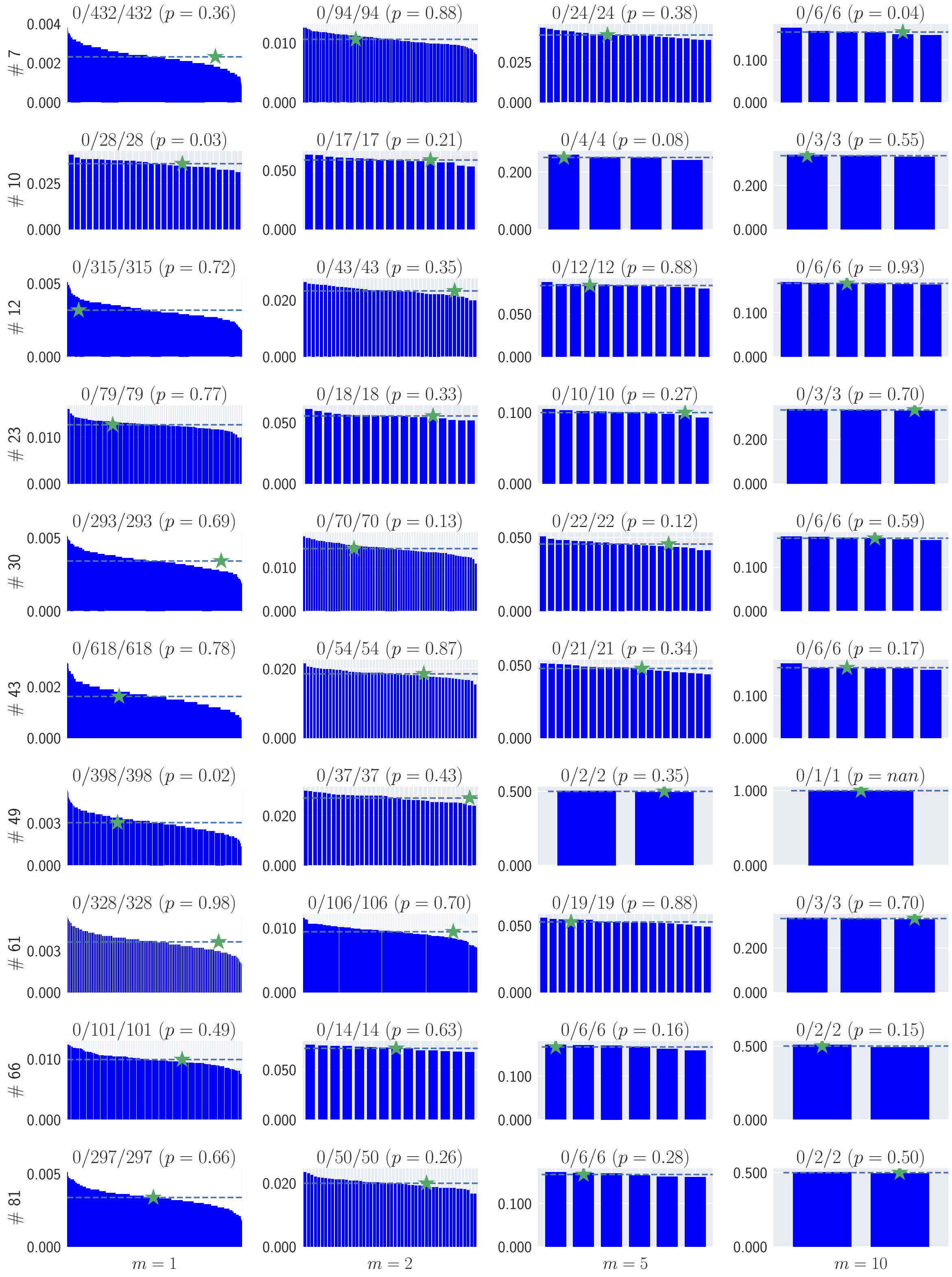

Figure S8 Rejection sampling results. Each plot shows the relative frequency of correct solutions (satisfying (SC)) output by our rejection sampling procedure (blue bars), with the simulated solution indicated by '⋆'. Dashed line indicates the expected relative frequency in the case of uniformity. The title of each plot lists the number of recovered correct solutions, the total number of correct solutions and the p-value of the chi-squared test of uniformity.

Author details
[1]Department of Computer Science, University of Illinois at Urbana-Champaign, Champaign, IL 61801, USA. [2]Department of Bioengineering, University of Illinois at Urbana-Champaign, Champaign, IL 61801, USA.

References

1. Creignou, N., Hermann, M.: On #P completeness of some counting problems. Research Report RR-2144, INRIA (1993). https://hal.inria.fr/inria-00074528
2. El-Kebir, M., Satas, G., Oesper, L., Raphael, B.J.: Inferring the Mutational History of a Tumor Using Multi-state Perfect Phylogeny Mixtures. Cell Systems **3**(1), 43–53 (2016)
3. Jamal-Hanjani, M., Wilson, G.A., McGranahan, N., Birkbak, N.J., Watkins, T.B.K., Veeriah, S., Shafi, S., Johnson, D.H., Mitter, R., Rosenthal, R., Salm, M., Horswell, S., Escudero, M., Matthews, N., Rowan, A., Chambers, T., Moore, D.A., Turajlic, S., Xu, H., Lee, S.M., Forster, M.D., Ahmad, T., Hiley, C.T., Abbosh, C., Falzon, M., Borg, E., Marafioti, T., Lawrence, D., Hayward, M., Kolvekar, S., Panagiotopoulos, N., Janes, S.M., Thakrar, R., Ahmed, A., Blackhall, F., Summers, Y., Shah, R., Joseph, L., Quinn, A.M., Crosbie, P.A., Naidu, B., Middleton, G., Langman, G., Trotter, S., Nicolson, M., Remmen, H., Kerr, K., Chetty, M., Gomersall, L., Fennell, D.A., Nakas, A., Rathinam, S., Anand, G., Khan, S., Russell, P., Ezhil, V., Ismail, B., Irvin-sellers, M., Prakash, V., Lester, J.F., Kornaszewska, M., Attanoos, R., Adams, H., Davies, H., Dentro, S., Taniere, P., O'Sullivan, B., Lowe, H.L., Hartley, J.A., Iles, N., Bell, H., Ngai, Y., Shaw, J.A., Herrero, J., Szallasi, Z., Schwarz, R.F., Stewart, A., Quezada, S.A., Le Quesne, J., Van Loo, P., Dive, C., Hackshaw, A., Swanton, C., TRACERx Consortium: Tracking the Evolution of Non-Small-Cell Lung Cancer. New England Journal of Medicine **376**(22), 2109–2121 (2017)
